# Prevalence of *Strongyloides stercoralis* infection among African children: A systematic review and meta-analysis

**DOI:** 10.1371/journal.pntd.0014759

**Published:** 2026-10-07

**Authors:** Tadesse Hailu, Arancha Amor, Getaneh Alemu, Hiwot Tadesse, Veeranoot Nissapatorn, Hesham M. Al-Mekhlafi

**Affiliations:** 1 Department of Medical Parasitology and Entomology, College of Medicine and Health Sciences, Bahir Dar University, Bahir Dar, Ethiopia; 2 Reference and Research Laboratory in Parasitology, National Center for Microbiology, Instituto de Salud Carlos III, Madrid, Spain; 3 Mundo Sano Foundation, Madrid, Spain; 4 Futuristic Science Reach Center, School of Science, and World Union for Herbal Drug Discovery (WUHeDD), Walailak University, Nakhon Si Thammarat, Thailand; 5 Department of Parasitology, Faculty of Medicine, Universiti Malaya, Kuala Lumpur, Malaysia; Rio de Janeiro State University: Universidade do Estado do Rio de Janeiro, BRAZIL

## Abstract

*Strongyloides stercoralis* is a neglected parasite that causes strongyloidiasis. However, the true prevalence of strongyloidiasis is underreported due to low-sensitivity diagnostic methods. This systematic review assessed the prevalence of *S. stercoralis* among African children and its variation by diagnostic methods, countries, and study settings. All studies published in African countries on *S. stercoralis* prevalence up to the year 2025 in the PubMed, Google Scholar, Scopus, and ScienceDirect databases were included. Identification, screening, checking the eligibility, and including the relevant studies were done following the Preferred Reporting Items for Systematic Reviews and Meta-Analyses (PRISMA). Out of 651 articles screened, 27 studies involving 16,358 children were included in the systematic review. The pooled prevalence of *S. stercoralis* among African children was 14% using a random-effect model. The highest pooled prevalence of *S. stercoralis* was recorded at 27% in Côte d’Ivoire, followed by 26% in Ethiopia. A high prevalence (36%) of *S. stercoralis* was obtained by a combination of PCR, culture, and microscopic diagnostic methods. This review indicated that *S. stercoralis* prevalence in African children is considerable and likely underestimated due to low-sensitivity diagnostics. Therefore, further studies using sensitive diagnostic approaches are urgently needed to determine the true burden of strongyloidiasis.

## Author summary

Strongyloides stercoralis remains a neglected soil-transmitted helminth with limited data on its burden among children in Africa. This systematic review and meta-analysis aimed to estimate the pooled prevalence and assess diagnostic approaches across the continent. A comprehensive search of PubMed, Scopus, Google Scholar, and ScienceDirect identified 651 records, of which 27 studies met the inclusion criteria. Using a random-effects model, the pooled prevalence of *S. stercoralis* infection was high with substantial heterogeneity across studies. Lower estimates were reported in studies using less sensitive diagnostic methods such as direct microscopy and formol-ether concentration. In contrast, studies employing combined techniques, including PCR and culture methods, reported markedly higher detection rates. These findings highlight the underestimation of infection due to diagnostic limitations and emphasize the need for improved surveillance, sensitive diagnostic tools, and targeted control strategies for children in endemic settings.

## Introduction

*Strongyloides stercoralis,* which causes strongyloidiasis, is one of the most neglected soil-transmitted helminths (STHs). It has a cosmopolitan distribution in tropical and subtropical regions [1]. An estimated 613.9 million *S. stercoralis* infections occur globally, of which 76.1% of these infections are concentrated in the Southeast Asia, Western Pacific, and African regions [2].

The risk of acquiring *S. stercoralis* infection is higher in rural dwellers and among population groups practicing open defecation and poor sanitation [3]. Since children are frequently playing with soil, they are at higher risk of *S*. *stercoralis* infection [4–5]. Malnutrition, a common problem in the African children [6], and an impaired immune system may contribute to the high prevalence of *S. stercoralis* infection [7].

Individuals infected with *S. stercoralis* may exhibit a spectrum of clinical manifestations, ranging from asymptomatic to life-threatening clinical symptoms depending on their immune status [8]. The larval stages cause clinical manifestations in the skin and lungs, whereas the adult worms reside in the small intestine, where intestinal manifestations occur [9], with major symptoms including skin irritation at the site of larval penetration, dry cough and/or tracheal irritation, and gastrointestinal symptoms [10]. In immunocompromised individuals, the infection may progress to hyperinfection syndrome or disseminated strongyloidiasis, which can be severe and life-threatening [11].

Among the 82 studies in Africa, the prevalence of strongyloidiasis ranged from 0.1% in a study conducted in Sudan to 27.1% in Côte d’Ivoire. The true prevalence of *S. stercoralis* is unknown, and the prevalence ranges from 0.2 to 27% in the African children since the majority of *S. stercoralis* infections are diagnosed by using direct smear microscopy (DSM) and formalin-ether concentration (FEC) techniques [12]. However, recent studies from different African countries reported that the prevalence of *S*. *stercoralis* infection among children using highly sensitive diagnostic methods is relatively high [13–15]. Indeed, sensitive diagnostic methods like the Baermann concentration technique (BCT), agar plate culture (APC), and quantitative polymerase chain reaction (qPCR) and their combinations are not used as a routine diagnostic approach in Africa [16,17]. In this review, we hypothesized that the pooled prevalence of *S. stercoralis* infection among African children is underestimated in the published literature due to the widespread use of low-sensitivity diagnostic methods and that prevalence estimates differ significantly across countries, diagnostic methods, and study settings. Therefore, the aim of this systematic review and meta-analysis was to estimate the pooled prevalence of *S. stercoralis* infection among children in Africa and to compare prevalence estimates according to country, diagnostic method, and study setting.

## Materials and methods

### Search strategy

The databases Google Scholar, PubMed, Scopus, and ScienceDirect were searched for studies written in English from the year 1966 up to 2025. The systematic review was carried out in accordance with the Preferred Reporting Items for Systematic Reviews and Meta-Analyses (PRISMA) guidelines (Fig 1). The review protocol was prospectively registered in the International Prospective Register of Systematic Reviews (PROSPERO) under the registration number CRD420261325085.

**Fig 1:**
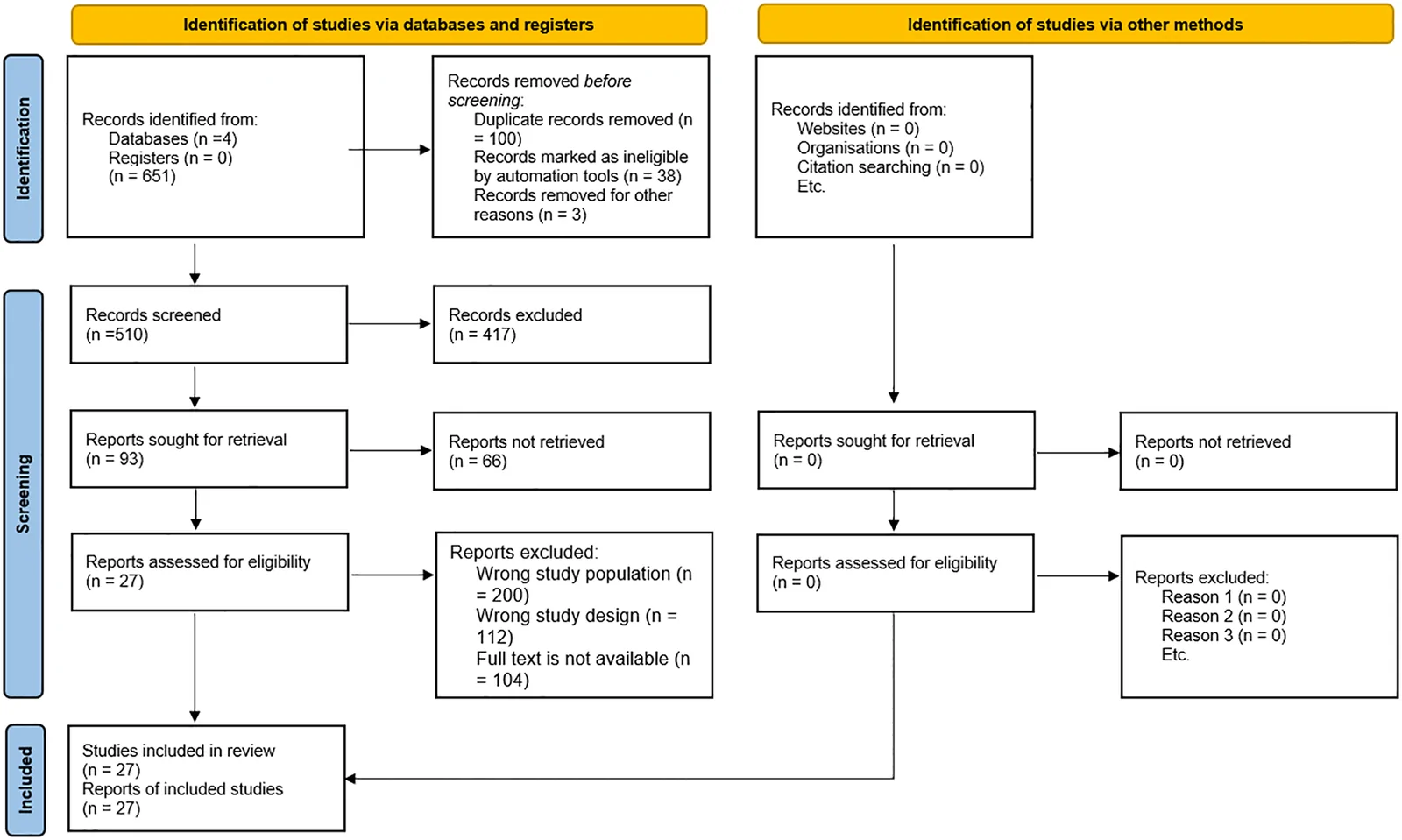
Overview of search methods articles inclusion and exclusion criteria.

Keywords used in the search were “Prevalence AND *Strongyloides* AND Children AND Africa or *Strongyloides stercoralis* AND Children AND African countries. Mesh term for PubMed: ((((“*Strongyloides stercoralis*/drug effects”[Mesh] OR “*Strongyloides stercoralis*/isolation and purification”[Mesh] OR “*Strongyloides stercoralis*/parasitology”[Mesh] OR “*Strongyloides stercoralis*/pathogenicity”[Mesh]) OR *S. stercoralis* OR *Strongyloides stercoralis* OR *Strongyloides* OR Strongyloidiasis) AND (“Prevalence”[Mesh] OR Prevalence OR epidemiology OR distribution OR survey)) AND ("Africa"[Mesh] or African countries)) AND (“Child”[Mesh] OR children OR kids OR schoolchildren). The search of studies was conducted in February 2026. Identification, screening, checking the eligibility, and the inclusion of the relevant studies were done following the preferred reporting items for systematic reviews and meta-analyses (PRISMA) (Fig 1). The articles extracted from the four databases were first screened to remove duplication. Then, the articles were screened by reading their title and abstracts. Finally, the full articles were screened, whether they investigated the prevalence of *S. stercoralis* among children or not.

### Selection criteria

Studies were considered eligible for inclusion if they met the following criteria: (i) observational study design; (ii) conducted in African countries; (iii) involved examination of children’s stool or blood samples; and (iv) reported the prevalence of *S. stercoralis* infection or provided sufficient data to calculate it (i.e., sample size, number of positive cases, and ages <14 years).

Studies were excluded if they focused on *S. stercoralis* infections in adult humans, animals, soil, or vegetables; involved foreign populations in African countries or imported cases; had undefined study populations or inappropriate age categorization; reported only *S. stercoralis*-positive cases; focused on method comparisons; or were detected by Kato-Katz; or were case reports, cohort studies, duplicate publications, or review articles conducted in African countries. The suitability of all studies according to the defined criteria above was judged independently by two different authors. Any difference in judgement was resolved by discussion among the authors. In each selected article, the following information was extracted: authors, year, country, sample size, the number of infected individuals, types of diagnostic methods used, and the study setting.

### Study quality assessment

The quality of the included studies was evaluated using the Joanna Briggs Institute (JBI) checklist for prevalence studies [18]. This tool consists of nine items, including (i) whether the sample frame was appropriate to represent the target population; (ii) if participants were sampled appropriately; (iii) adequacy of sample size; (iv) detailed description of study participants and setting; (v) data analysis covering the identified sample; (vi) use of valid methods to identify the condition; (vii) standardized and reliable measurement of the condition for all participants; (viii) appropriate statistical analysis; and (ix) sufficient response rate. Each item was scored as 1 for “yes” and 0 for “not reported.” The total “yes” scores for each study were calculated and categorized as follows: low quality (high risk of bias, score < 5), moderate quality (moderate risk of bias, score 5–7), and high quality (low risk of bias, score 8–9). Studies rated as low quality were still included in the final analysis.

The meta-analysis was performed using STATA version 18 software (StataCorp LLC, Texas, USA). The pooled prevalence of *S. stercoralis* infection among children at a 95% confidence interval (CI) in African countries was calculated using a random-effects model. The pooled prevalence of *S. stercoralis* infection among children by country and diagnostic methods was also calculated in the subgroup analysis. The forest plot was reported, and separate meta-analyses were performed to evaluate the effect of diagnostic methods with *S. stercoralis* infection among children. Heterogeneity (the difference between studies) by country, diagnostic methods, and study site was assessed using Cochran’s (Q) value, *P-value,* and I^2^ and visual inspection of the forest plot. The level of significance for all tests was *P < 0.001*. Publication bias that occurs in published studies was checked by considering effect size symmetry on a funnel plot (a scatter plot of estimates). Absence of bias is presented by a shape like a funnel. Observational studies were also initially considered as low-certainty evidence.

## Result

The databases Google Scholar (n = 334), PubMed (n = 189), Scopus (n = 102), and ScienceDirect (n = 26) were searched for studies written in English from the year 1966 up to 2025. A total of 651 articles were retrieved in 8 African countries, which had *S. stercoralis* research reports and fulfilled the inclusion criteria. In the 27 studies, 16358 children under 14 years old of age were included. Of those 27 studies, 9 (33.3%) were from Nigeria, followed by 6 (22.2%) from Ethiopia, which accounted for 3842 (23.5%) and 2762 (16.9%) participants, respectively. The prevalence ranges from 0.07% in Angola to 48.5% in Ethiopia (Table 1; Fig 2).

**Table 1:** Prevalence of *Strongyloides stercoralis* among children in African countries between 2010 - 2024.

Reference	Country	Diagnostic method	Age (years)	Data collection time	Study setting	Sample size	Strongyloides Positive n (%)
Soultani *et al* 2024 [19]	Angola	qPCR	8-11	2021	School	3063	145 (4.7)
Dacal *et al*, 2018 [20]	Angola	PCR	5-14	Jan-June, 2015	school	351	75 (21.4)
de Alegrıa *et al*, 2017 [13]	Angola	FEC, BCT	5-14	Jan-May 2015	school	230	28 (12.2)
Bocanegra *et al,* 2015 [21]	Angola	FEC	9-10	Mar 2013-Feb 2014	school	1425	1 (0.07)
Glinz *et al*., 2010 [22]	Côte d’Ivoire	BCT, APC	6-14	Apr–May 2008	School	251	68 (27.1)
Abera *et al*., 2013 [23]	Ethiopia	FEC	7-14	March 2011	School	788	27 (3.4)
Amor *et al.,* 2016 [24]	Ethiopia	FEC, BCT, PCR	5-14	Oct to Nov 2013	School	396	82 (20.7)
Nyantekyi *et al*., 2010 [25]	Ethiopia	FEC	≤5	Jun 2007	Community	288	38 (13.2)
Aramendia *et al.,* 2020 [14]	Ethiopia	FEC, BCT, qPCR	6-14	Feb to June 2016	Community	301	146 (48.5)
Woyneshet *et al.,* 2021 [26]	Ethiopia	BCT	≤14	August 12–31/2019	Community	145	43 (29.7)
Jember *et al,* 2022 [17]	Ethiopia	FEC, STST, BCT, APC, qPCR	6-14	Apr to Dec 2019	School	844	329 (39.0)
Forson *et al.,*2017 [27]	Ghana	DSM, FEC	2-9	Mar 2016-July 2016	School	300	1 (0.3)
Sam *et al.,* 2018 [28]	Ghana	DSM, FEC	5-14	Oct 2010-Mar 2011	School	394	20 (5.1)
Ferreira *et al*., 2020 [29]	Mozambique	DSM, FEC	≤5	2012	Hospital	831	34 (4.1)
Muadica *et al*., 2021 [30]	Mozambique	PCR	3‒14	Oct 2017-Feb 2019	School + Clinic	1093	232 (21.2)
Sunday *et al*., 2022 [31]	Nigeria	FEC	1-12	Apr2015-Mar2016	Community	496	30 (6.0)
Evbuomwan *et al.,* 2021 [32]	Nigeria	FEC	6-15	Aug-Oct 2018	School	248	85 (34.3)
Nwankwo *et al*., 2022 [33]	Nigeria	DSM, FEC	6-15	June-Sep 2017	School	453	9 (2.0)
Nwadiuto *et al*., 2021 [34]	Nigeria	FEC	5-12	May to July 2018	School	150	13 (8.7)
Oniya *et al.,* 2021 [35]	Nigeria	FEC	≤14	Jan-Sep 2012	School	298	59 (19.8)
Uhuo *et al*., 2011 [36]	Nigeria	FEC	4-14	NA	School	800	4 (0.5)
Simon-Oke *et al*., 2014 [37]	Nigeria	DSM, FEC	5-14	NA	School	180	23 (12.8)
Olusegun *et al*., 2014 [38]	Nigeria	DSM, FEC	3-14	NA	School	162	6 (3.7)
Esiet and Edet, 2017 [39]	Nigeria	DSM, FEC	6-13	NA	School	1055	46 (4.4)
Ferreira *et al.,* 2015 [40]	Sao Tome & Principe	DSM, FEC	1-5	May 2011	Community	444	11 (2.5)
Salim *et al.,* 2014 [41]	Tanzania	BCT	3-10	Jan 2011-Nov 2012	Community	1033	71 (6.9)
Eltantawy *et al*., 2021 [15]	Tanzania	BCT	8-12	May-June 2014	School	339	93 (27.4)
Total						16358	1574 (9.6)

* DSM = Direct smear microscopy, FEC = Formalin-ether concentration, BCT = Baermann concentration technique, APC = Agar plate culture, qPCR = Quantitative polymerase chain reaction, PCR = Polymerase chain reaction, STST = Spontaneous tube sedimentation technique, qPCR = Quantitative polymerase chain reaction.

**Fig 2:**
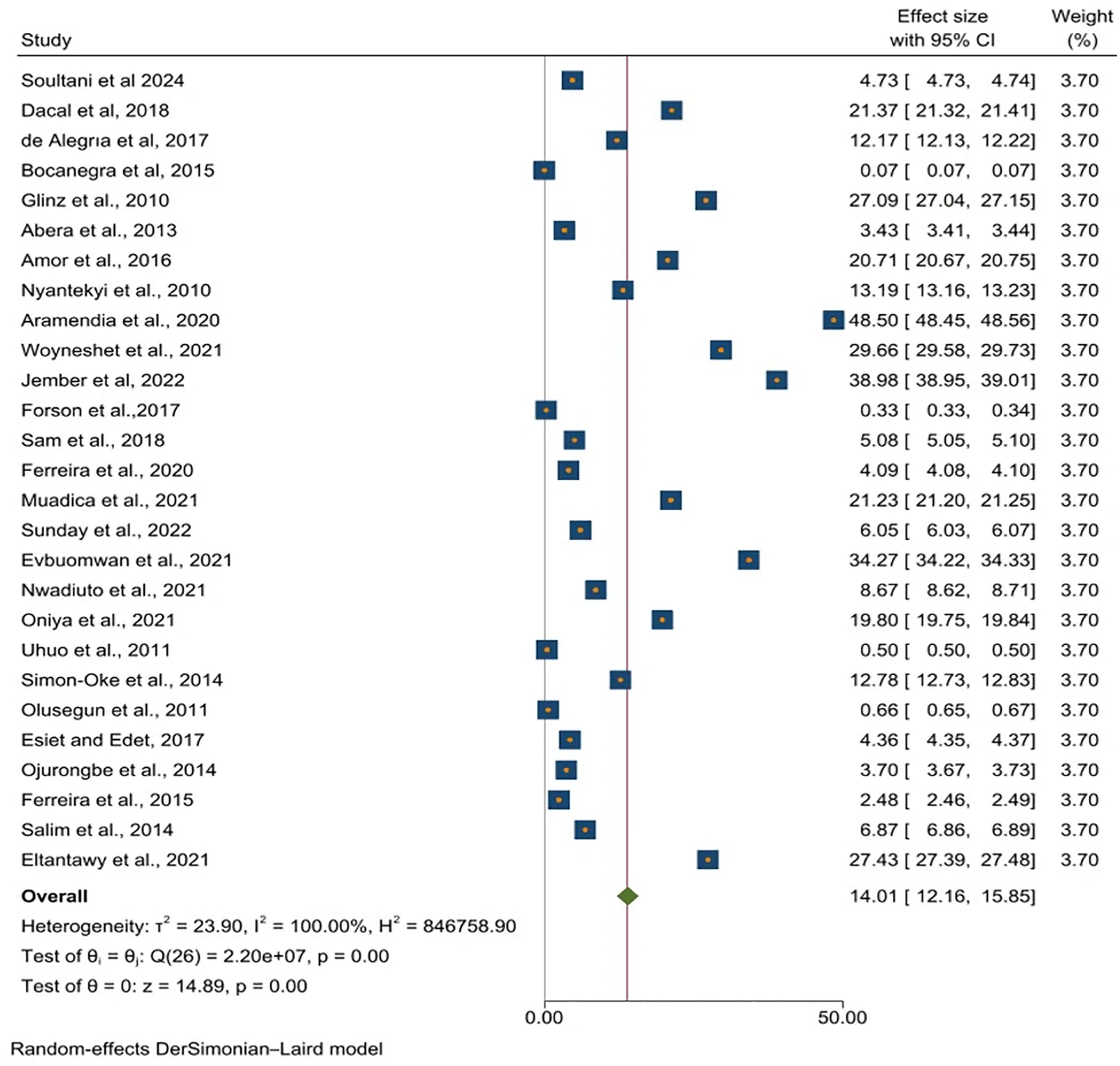
The pooled prevalence of *S. stercoralis* infection among African children, 2026.

Among the 27 studies, only 11 (40.7%) studies were conducted using highly sensitive diagnostic methods, including BCT, APC, qPCR, or a combination of them [13–15,17,19,22,24,26,30,41]. Of which four studies were conducted in Ethiopia using one of the above diagnostic tests or a combination of them [14,17,24,26] followed by three studies in Angola [13,17,19] and two studies in Tanzania [15,41] (Table 1).

The majority of the studies in Africa, 16 (59.3%), were conducted by FEC or DSM or a combination of these two methods, which have a very low sensitivity in the detection of *S. stercoralis* [12]. All 9 (33.3%) studies in Nigeria were conducted by FEC or a combination of DSM and FEC diagnostic methods [31–39]. Lower prevalence of *S. stercoralis* among African children was reported in 0.07% in Angola [20], 0.3% in Ghana [27], and 0.5% in Nigeria [36] (Table 1; Fig 2).

The forest plots (Fig 2) indicated the pooled prevalence of *S. stercoralis* infection among African children in 27 studies was 14.0% (95% CI: 12.2-15.9%) using a random-effects model. The overall heterogeneity of studies across African countries was (Q = 2.20e + 07, I^2^ = 100%, *P ≤ 0.001*) (Fig 2).

In the subgroup analysis, a significant difference was observed between countries (Q = 720,132.7, P < 0.001) based on Cochran’s Q test. The highest pooled prevalence (27%) of *S. stercoralis* was reported in Côte d’Ivoire, followed by Ethiopia (26%) and Tanzania (17%) (Fig 3). In contrast, the lowest pooled prevalence was reported in São Tomé and Príncipe (2%), followed by Ghana (3%). The highest detection rate (36%) was observed when PCR was combined with culture and microscopy, followed by culture alone (23%) (Fig 3).

**Fig 3:**
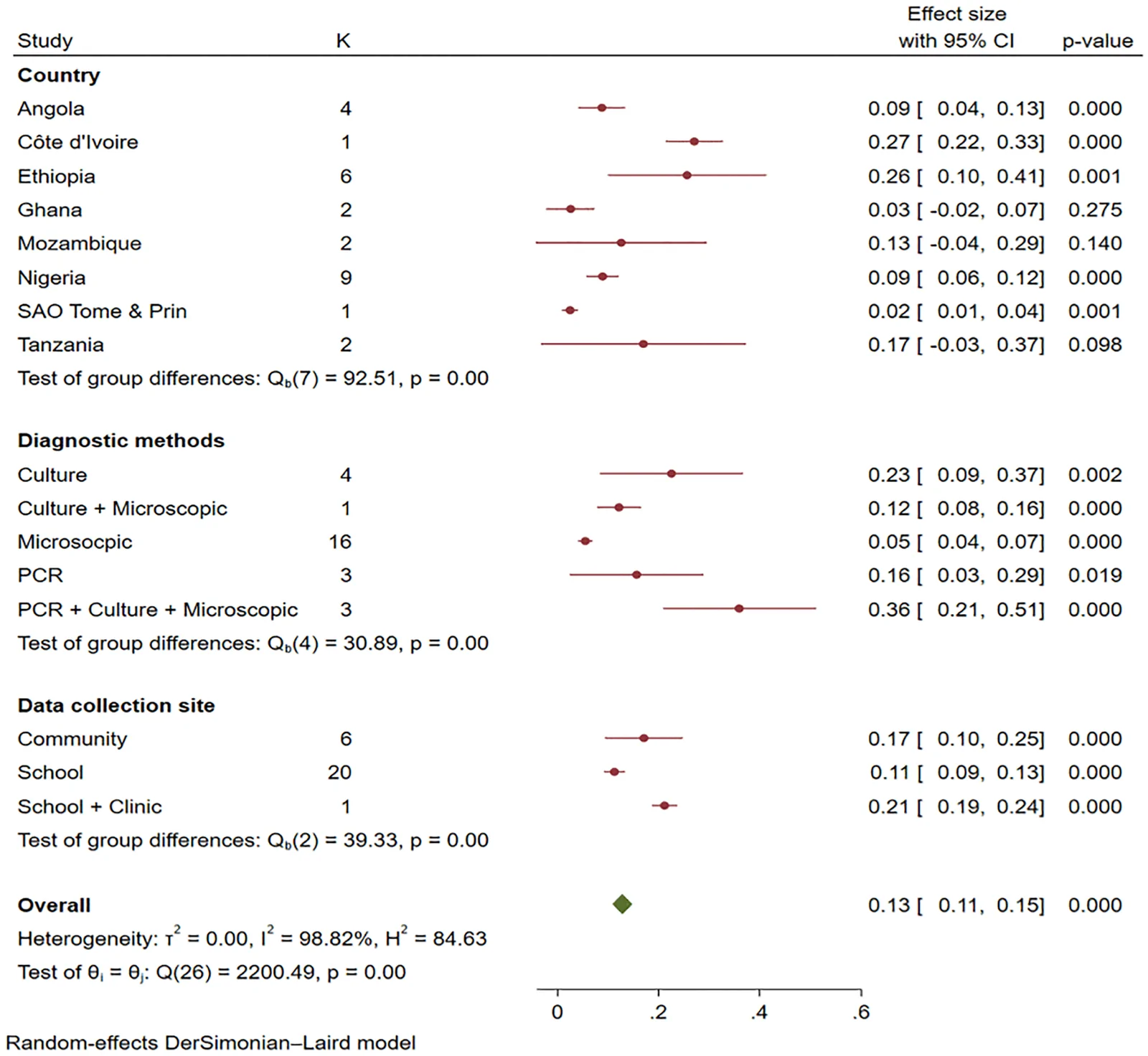
The subgroup analysis of *S. stercoralis* infection by country, diagnostic methods and data collection sites, 2026.

A significant difference in *S. stercoralis* prevalence was also observed between data collection sites (Q = 87.6, P < 0.001). The highest prevalence was reported among samples collected in school and clinic settings (21%), followed by community-based samples (17%) (Fig 3).

The funnel plot indicated that the included studies were roughly symmetrically distributed around the pooled prevalence estimate of strongyloidiasis. This pattern suggested that there was no clear evidence of publication bias in the meta-analysis (Fig 4).

**Fig 4:**
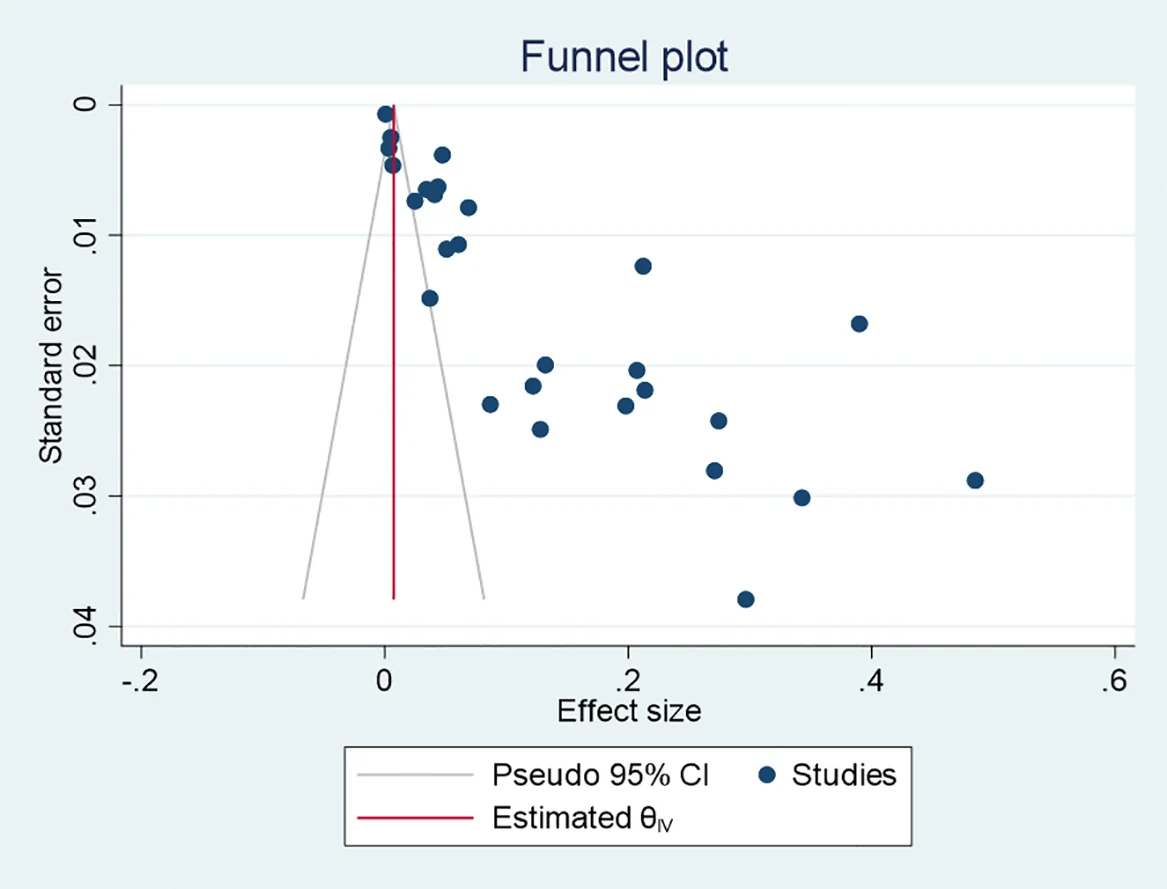
Funnel plot displaying *S. stercoralis* prevalence data for all included publications, 2026.

## Discussion

Estimating the true prevalence of *S. stercoralis* infection among children in Africa is challenging due to limited studies in each country, application of low-sensitivity diagnostic methods, and lack of specific diagnostic methods. Although DSM or FEC diagnostic methods are widely used detection methods for helminths from fecal samples, they underdiagnose *S. stercoralis* [12,42]. Our findings demonstrated that the prevalence conducted with inadequate methods, including DSM, FEC methods, or their combinations, provided unreliable figures among children living on the African continent.

On the other hand, studies conducted with highly sensitive diagnostic methods like APC, BCT, PCR, or a combination of these methods provided a high prevalence of *S. stercoralis* among the African children [13,14,17,20,22,24,43,44]. However, the sensitivity of such methods is not always optimal, particularly when performed on a single stool specimen or diarrheic stool, which may underestimate the true prevalence. Therefore, there is a need to define a standard protocol for the diagnosis of *S. stercoralis* to get a valid result that helps in mapping *S. stercoralis* in endemic areas in Africa.

The prevalence of *S. stercoralis* is also varied among countries, as observed in the subgroup analysis. For instance, the relatively high prevalence in Ethiopia might be due to the application of a more sensitive diagnostic method or combination of diagnostic methods, the presence of risk factors for the transmission, and the endemicity of *S. stercoralis* in the area [3]. On the other hand, the lowest prevalence was reported in São Tomé and Príncipe and Ghana (Fig 3), which could be due to the application of low-sensitivity diagnostic methods, including DSM and FEC [25,26,38].

The low *S. stercoralis* prevalence report in this study was reported due to the utilization of DSM, FEC or a combination of these methods. This showed that both DSM and FEC, or their combinations are not sensitive enough to detect *S. stercoralis* infection due to a small amount of stool sample, low larval load in stool and intermittent excretion of *S. stercoralis* larvae in stool [45,46].

Besides diagnostic methods, these variations may be due to various environmental conditions, sanitation, socioeconomic status, and public health interventions that may affect the transmission of the causative agents [3].

Variation was also observed between different sampling settings, with higher prevalence observed in the community compared to school-based studies (Fig 3). This variation may be attributed to the inclusion of non-school-attending and higher-risk children’s differences in exposure and hygiene practices, as well as the protective effect of school-based health interventions such as deworming and health education.

The risk of *S. stercoralis* infection among children is influenced by age, environmental exposure, and behavioral factors. Differences in age groups, living conditions, hygiene practices, sanitation infrastructure, access to safe water, and socioeconomic status among included studies may contribute to variations in prevalence estimates [3]. Older children may have greater cumulative exposure through outdoor activities and soil contact, while younger children may be influenced by household-related exposures. Variations in lifestyle factors, footwear use, rural or urban residence, and environmental conditions across African settings should be considered when interpreting heterogeneity and designing context-specific surveillance and control strategies aligned with WHO recommendations [3,47].

This review has several limitations, including the predominance of cross-sectional studies, methodological heterogeneity in study designs, geographic regions, diagnostic methods, and generally low-sensitivity diagnostic techniques. Additionally, the restriction to English-language publications, the possible omission of grey literature, the absence of automation tools during study selection, and the uneven distribution of studies across African countries may have affected the completeness, generalizability, and accuracy of the pooled prevalence estimates.

## Conclusion

Strongyloides stercoralis infection among children still ranks as one of the most neglected tropical infections that needs more attention from policymakers, especially in the continent of Africa, where open defecation and poor personal hygiene are still prevalent. A substantially higher prevalence of strongyloidiasis was detected using combined diagnostic approaches, highlighting that the true burden of strongyloidiasis is likely underestimated when relying on conventional, low-sensitivity techniques. Thus, we urge researchers to strive towards establishing standard procedures for diagnosing strongyloidiasis and support the implementation of the WHO Guideline on preventive chemotherapy for public health control of strongyloidiasis to strengthen surveillance, diagnosis, and control efforts in endemic African settings.

## Supporting information

S1 PRISMA Checklist PRISMA 2020 Checklist. (DOCX)
