# Supplementary material for: Prevalence of Strongyloides stercoralis infection among African children: A systematic review and meta-analysis
Source: PLoS Negl Trop Dis. 2026 Oct 7;20(10):e0014759. doi: 10.1371/journal.pntd.0014759 (PMC13667923; doi:10.1371/journal.pntd.0014759)
Supplement: Supplementary file 1 [file pntd.0014759.s002.pdf]

Manuscript Title: Prevalence of *Strongyloides stercoralis* Infection among African Children: A Systematic Review and Meta-Analysis

Summary of the research

The manuscript addresses a critical public health gap by pooling data on the prevalence of *Strongyloides stercoralis* among African children up to 2025. The study appropriately highlights that widespread reliance on low-sensitivity diagnostic tools (DSM, FEC) significantly underestimates the true disease burden. The systematic integration of 27 studies comprising 16,358 children provides a valuable epidemiological baseline. However, the text requires substantial structural, statistical, and grammatical refinements before it is suitable for publication.

Minor Revisions and Methodological Issues

1 Discrepancy in Data and Text Presentation in Settings Findings: In the Result section, the text states: "The highest prevalence was reported among samples collected in school and clinic settings (21%), followed by community-based samples (17%)." However, in the Discussion section, the text contradicts this: "There was also observed a variation in the study results between different sampling settings, with higher prevalence observed in the community compared to school-based studies." Resolve this contradiction to ensure data integrity.

2 Clarification of Acronyms and Diagnostic Subgroups
   i. Undefined Acronyms: Even though the diagnostic methods introduced as acronyms were defined in Table 1, there should be prior definitions upon first mention for BCT, APC, FEC, and DSM (e.g., Formol-Ether Concentration, Direct Stool Microscopy) in the text.
   ii. Subgroup Definition: Labelling methods as "better sensitive diagnostic methods" is subjective. Clearly specify what qualifies as "advanced/high-sensitivity" (such as molecular PCR, agar plate culture) versus "traditional/low-sensitivity" in both the methodology and results sections.

3 Interpretation of Extreme Heterogeneity
   i. The meta-analysis shows an I^2 of 100% ($Q = 2.20e+07$, $P \leq 0.001$). An I^2 at this level signifies complete heterogeneity, suggesting that a single pooled prevalence estimate of 14.0% might not be clinically useful.
   ii. The Discussion section should expand on this limitation. Emphasise that the pooled figure is a mathematical average across highly diverse study designs, geographic features, and diagnostic sensitivities, rather than a uniform representation of the continent.

4 Corrections for grammatical errors and syntax issues
   i. Third-Person Reference Issue: In the Discussion section (paragraph 1), the text states: "The authors clearly demonstrated that..." Since this is the authors' own manuscript,

rephrase this to avoid writing in the third person about your own work (e.g., "This review demonstrates that..." or "Our findings demonstrate that...").

ii. Awkward phrasing
Current: "In the 27 studies, 16,358 children who were younger than 14 years old were included."
Correction: "The 27 included studies comprised 16,358 children under 14 years of age."
Current: "There was also observed a variation..."
Correction: "Variation was also observed..."

Recommendation to the Editor

Decision: Minor Revision

The manuscript has significant epidemiological value and focuses on a neglected tropical disease. However, it cannot be accepted until the contradictions between the Results and Discussion sections are clearly resolved and the manuscript has been carefully copy-edited.
