# Supplementary material for: Prevalence of Strongyloides stercoralis infection among African children: A systematic review and meta-analysis
Source: PLoS Negl Trop Dis. 2026 Oct 7;20(10):e0014759. doi: 10.1371/journal.pntd.0014759 (PMC13667923; doi:10.1371/journal.pntd.0014759)
Supplement: Supplementary file 2 [file pntd.0014759.s001.docx]

| **Section and Topic** | **Item #** | **Checklist item** | **Location where item is reported** |
| --- | --- | --- | --- |
| **TITLE : Prevalence of *Strongyloides stercoralis* Infection among African Children: A Systematic Review and Meta-Analysis** | | |  |
| Title | 1 | This report is a systematic review and meta-analysis that synthesizes available evidence on the prevalence of Strongyloides stercoralis infection among children in Africa. The study systematically identifies, appraises, and analyzes published research to estimate the pooled prevalence of infection and assess variations across different countries, study settings, and diagnostic methods. | Page 1, line 1 |
| **ABSTRACT** | | |  |
| Abstract | 2 | **Background:** *Strongyloides stercoralis* is a neglected parasite that causes strongyloidiasis. However, the true prevalence of strongyloidiasis is underreported due to low-sensitivity diagnostic methods. This systematic review assessed the prevalence of *S. stercoralis* among African children and its variation by diagnostic methods, countries, and study settings.  **Methods:** All studies published in African countries on *S. stercoralis* prevalence up to the year 2025 in the PubMed, Google Scholar, Scopus, and ScienceDirect databases were included. Identification, screening, checking the eligibility, and including the relevant studies were done following the Preferred Reporting Items for Systematic Re­views and Meta-Analyses (PRISMA). Publication bias was assessed using funnel plot symmetry and statistical tests.  **Results**: Out of 651 articles screened, 27 studies involving 16,358 children were included in the systematic review. The pooled prevalence of *S. stercoralis* among African children was 14% using a random-effect model. The highest pooled prevalence of *S. stercoralis* was recorded at 27% in Côte d’Ivoire, followed by 26% in Ethiopia. A high prevalence (36%) of *S. stercoralis* was obtained by a combination of PCR, culture, and microscopic diagnostic methods. This review indicated that *S. stercoralis* prevalence in African children is considerable and likely underestimated due to low-sensitivity diagnostics. Therefore, further studies using sensitive diagnostic approaches are urgently needed to determine the true burden of strongyloidiasis.  Registration: PROSPERO registration number: CRD420261325085.  Funding: “No specific funding was received for this study.” | Page 1-2, line 12-28 |
| **INTRODUCTION** | | |  |
| Rationale | 3 | The true burden of *Strongyloides stercoralis* infection among African children remains poorly understood despite the parasite being one of the most neglected soil-transmitted helminths in tropical and subtropical regions. Existing studies conducted across African countries have reported highly variable prevalence estimates, largely due to differences in geographic settings, study populations, and particularly the diagnostic methods employed. Most epidemiological studies in Africa rely on conventional low-sensitivity techniques such as direct smear microscopy and formalin-ether concentration methods, which are known to underestimate the prevalence of strongyloidiasis. In contrast, studies using more sensitive diagnostic approaches, including Baermann concentration, agar plate culture, and molecular methods, have demonstrated substantially higher detection rates. Accurate estimates of the burden of *S. stercoralis* infection are essential for informing public health policies, improving diagnostic strategies, and guiding prevention and control programs targeting children, who are among the most vulnerable population groups. However, no comprehensive systematic review and meta-analysis has specifically synthesized the prevalence of *S. stercoralis* infection among African children across different countries, diagnostic methods, and study settings. Therefore, this systematic review and meta-analysis was conducted to provide pooled prevalence estimates and to evaluate variations in prevalence according to diagnostic approaches and epidemiological settings in Africa. | Page 3-4, line 60-71 |
| Objectives | 4 | This systematic review and meta-analysis aimed to estimate the pooled prevalence of Strongyloides stercoralis infection among children in African countries. In addition, the review assessed variations in prevalence according to country, diagnostic method, and study setting. The review also evaluated the impact of diagnostic approaches on the reported prevalence of strongyloidiasis among African children. | Page 4, line 71-73 |
| **METHODS** | | |  |
| Eligibility criteria | 5 | Studies were included in this systematic review and meta-analysis if they were observational in design (including cross-sectional and baseline survey studies), conducted in African countries, and involved children aged below 14 years. Eligible studies were required to examine stool or blood samples for the detection of Strongyloides stercoralis infection and to report the prevalence of infection or provide sufficient data (sample size and number of positive cases) to allow prevalence calculation.  Studies were excluded if they focused on adult populations, animals, soil, or environmental samples, or if they reported imported cases or non-African resident populations. Studies were also excluded when age-specific data for children could not be extracted, when only positive cases were reported without denominators, or when they were methodological papers focused on diagnostic comparisons without prevalence estimates. In addition, case reports, review articles, duplicate publications, and studies using Kato-Katz alone for S. stercoralis diagnosis were excluded. | Page 5, line 94-102 |
| Information sources | 6 | Electronic databases searched included PubMed, Scopus, ScienceDirect, and Google Scholar. In addition, reference lists of all eligible articles and relevant review papers were manually screened to identify additional studies that might have been missed during the electronic search. No restriction was placed on publication year during the initial search.  The search strategy was implemented in February 2026, and each database was last searched on 28 February 2026. Google Scholar was also used to identify grey literature and additional relevant publications not indexed in the major databases. No separate trial registers were available for observational prevalence studies; therefore, no clinical trial registries were searched. All retrieved records from each source were combined, and duplicates were removed prior to screening. | Page 4, line 76-89 |
| Search strategy | 7 | For PubMed, a combination of Medical Subject Headings (MeSH) terms and free-text keywords was applied as follows:  ((((“Strongyloides stercoralis/drug effects”[Mesh] OR “Strongyloides stercoralis/isolation and purification”[Mesh] OR “Strongyloides stercoralis/parasitology”[Mesh] OR “Strongyloides stercoralis/pathogenicity”[Mesh]) OR “Strongyloides stercoralis” OR “S. stercoralis” OR “Strongyloides” OR “strongyloidiasis”) AND (“Prevalence”[Mesh] OR prevalence OR epidemiology OR distribution OR survey)) AND (“Africa”[Mesh] OR Africa OR African countries)) AND (“Child”[Mesh] OR child* OR children OR schoolchildren OR kids).  For Scopus, ScienceDirect, and Google Scholar, the following keyword-based search strategy was used:  (“Strongyloides stercoralis” OR “Strongyloides” OR “strongyloidiasis”) AND (prevalence OR epidemiology OR distribution OR survey) AND (children OR child OR schoolchildren OR kids) AND (Africa OR “African countries”). | Page 4, line 82-9 |
| Selection process | 8 | Two independent reviewers conducted the study selection process. All records identified through database searching were first imported into a reference management software, and duplicate entries were removed. The titles and abstracts of the remaining studies were then screened independently by the two reviewers to assess eligibility based on the predefined inclusion criteria.  Full-text articles of potentially relevant studies were subsequently retrieved and independently assessed for eligibility by the same two reviewers. Each report was evaluated against the inclusion and exclusion criteria, and reasons for exclusion at the full-text stage were recorded. Any disagreements between the two reviewers during both the screening and eligibility assessment stages were resolved through discussion and consensus; when necessary, a third reviewer was consulted to reach a final decision. All screening and selection steps were conducted manually to ensure accuracy and consistency in applying the eligibility criteria. | Page 4-5, line 90-94 |
| Data collection process | 9 | Specify the methods used to collect data from reports, including how many reviewers collected data from each report, whether they worked independently, any processes for obtaining or confirming data from study investigators, and if applicable, details of automation tools used in the process. | Page 4-5, line 76-94 |
| Data items | 10a | The primary outcome of this systematic review and meta-analysis was the prevalence of *Strongyloides stercoralis* infection among children in African countries, defined as the proportion of children who tested positive for the parasite out of the total number of children examined in each study. This was extracted directly from each eligible study based on reported positive cases and sample size.  Secondary outcomes included variations in prevalence estimates across subgroups, specifically by:   - Geographical location (country-level prevalence estimates) - Diagnostic method used (e.g., direct smear microscopy, formalin-ether concentration, Baermann technique, agar plate culture, and molecular methods such as PCR or combined diagnostic approaches) - Study setting (community-based versus institution-based studies)   For each included study, all available results corresponding to the prevalence of *S. stercoralis* infection among children were extracted. Where studies reported multiple time points or multiple diagnostic methods, the data corresponding to the primary reported prevalence for the study population of children (<14 years) were prioritized. If more than one diagnostic method was used within the same study, each method-specific prevalence was extracted separately and included in subgroup analyses rather than selecting a single estimate. This approach ensured that all relevant outcome data compatible with the review objectives were systematically captured and appropriately synthesized. |  |
|  | 10b | Data were also extracted on several study-level and participant-related variables to contextualize the prevalence estimates and facilitate subgroup analyses. These included: first author’s name, year of publication, country where the study was conducted, study setting (community-based or institution-based), study design (cross-sectional or baseline survey), sample size of children examined, number of S. stercoralis positive cases, and diagnostic methods used for detection. Additional variables included the age range of participants (restricted to children under 14 years), and where available, sex distribution of participants.  Information on study quality was also recorded based on the Joanna Briggs Institute (JBI) checklist assessment scores. Where reported, funding sources and conflict of interest statements were also extracted to assess potential sources of bias.  When data were missing or unclear, the following assumptions and approaches were applied: if studies did not explicitly state the age range but clearly indicated school-aged or pediatric populations, they were included if the context confirmed inclusion of children under 14 years. If diagnostic methods were described inconsistently, they were classified according to the most detailed method reported or grouped under “unclear/mixed methods” where clarification was not possible. When funding information was not reported, it was recorded as “not stated.” Similarly, if sex distribution or other participant characteristics were not available, these variables were treated as missing data and were not included in subgroup analyses. In cases of incomplete prevalence data, studies were excluded unless sufficient information (sample size and number of positives) allowed calculation of prevalence estimates. |  |
| Study risk of bias assessment | 11 | The risk of bias in the included studies was assessed using the Joanna Briggs Institute (JBI) critical appraisal checklist for prevalence studies. This tool evaluates methodological quality across nine domains, including sampling strategy, sample size adequacy, participant description, validity of diagnostic methods, statistical analysis, and response rate. Each item was rated as “yes” (1) or “no/unclear/not reported” (0), and an overall quality score was calculated to categorize studies as low, moderate, or high quality (risk of bias).  The risk of bias assessment was conducted independently by two reviewers. Each study was appraised separately by both reviewers, and their assessments were compared for consistency. Any discrepancies between the reviewers were resolved through discussion and consensus. When agreement could not be reached, a third reviewer was consulted to make the final decision. No automation tools or machine learning-based systems were used in the risk of bias assessment process. All evaluations were performed manually to ensure careful interpretation of study methodologies and diagnostic approaches.Top of Form  Bottom of Form | Page 5-6, 110-120 |
| Effect measures | 12 | For the primary outcome, which was the prevalence of Strongyloides stercoralis infection among children, the effect measure used was prevalence proportion, expressed as the number of infected children divided by the total number of children examined in each study. All study-specific prevalence estimates were presented with their corresponding 95% confidence intervals (CI).  For the pooled analysis, the effect size was the overall (pooled) prevalence, calculated using a random-effects meta-analysis model and reported as a proportion with a 95% CI.  For subgroup analyses, the same effect measure (prevalence proportion with 95% CI) was used, stratified by country, diagnostic method, and study setting to allow comparison of differences between groups.  No comparative effect measures such as risk ratios, odds ratios, or mean differences were used, as the review focused exclusively on estimating and comparing prevalence rather than associations or treatment effects. | Page 6, 121-131 |
| Synthesis methods | 13a | Studies were considered eligible for each synthesis based on predefined grouping criteria aligned with the review objectives. After full-text screening and final inclusion, all studies were first tabulated in a standardized extraction sheet summarizing key characteristics, including country, sample size, study setting, diagnostic method, and reported prevalence.  For the primary synthesis, all studies that met the inclusion criteria and reported sufficient data to compute prevalence were included in the overall meta-analysis of Strongyloides stercoralis infection among children.  For subgroup syntheses, studies were categorized and assigned based on their reported characteristics. Specifically, studies were grouped according to: (i) country or geographical region for country-level pooled estimates, (ii) diagnostic method used for detection of S. stercoralis (e.g., microscopy-based methods, concentration techniques, and molecular methods) for method-specific pooled prevalence, and (iii) study setting (community-based versus institution-based) for setting-specific analyses.  Each study contributed to all relevant subgroup analyses if it reported the necessary information. When a study reported multiple diagnostic methods, each method-specific result was assigned to the corresponding subgroup, while ensuring that double-counting was avoided in the overall pooled estimate by using a single overall prevalence per study. Decisions on study grouping were made independently by two reviewers during data extraction and verified during data synthesis to ensure consistency with the planned analytical framework. | Page 6, 121-131 |
|  | 13b | Prior to analysis, all extracted data were checked for completeness, consistency, and accuracy. Prevalence data were standardized by calculating the proportion of *Strongyloides stercoralis*–positive cases out of the total number of children examined for each study. Where confidence intervals were not reported in the original studies, they were calculated using standard binomial approximation methods. If studies reported data in subgroups without an overall combined estimate, subgroup data were combined where possible using standard aggregation formulas. When required information for prevalence calculation was missing or unclear and could not be obtained from the study authors or derived from available data, the study was excluded from quantitative synthesis but retained for qualitative description if relevant. All data transformations and preparations were performed prior to importing the dataset into STATA version 18 for meta-analysis.Top of Form  Bottom of Form | Page 6, 121-131 |
|  | 13c | The characteristics and results of individual studies were summarized using structured tables and graphical displays. Study characteristics, including author name, publication year, country, diagnostic method, study setting, sample size, and number of Strongyloides stercoralis–positive cases, were presented in tabular form to facilitate comparison across studies.  Forest plots were used to visually display study-specific prevalence estimates with their corresponding 95% confidence intervals, as well as the pooled prevalence estimates generated from the random-effects meta-analysis. Separate forest plots were also produced for subgroup analyses according to country, diagnostic method, and study setting to explore heterogeneity among studies.  A funnel plot was used to visually assess potential publication bias and small-study effects by examining the symmetry of prevalence estimates around the pooled effect size. Symmetrical distribution of studies around the pooled estimate was interpreted as indicating low likelihood of publication bias.  The Preferred Reporting Items for Systematic Reviews and Meta-Analyses (PRISMA Statement) flow diagram was used to illustrate the study selection process, including identification, screening, eligibility assessment, and final inclusion of studies in the review. | Page 6, 121-131 |
|  | 13d | The results were synthesized quantitatively using meta-analysis of proportions. A random-effects model was applied to estimate the pooled prevalence of Strongyloides stercoralis infection among children, as substantial clinical and methodological heterogeneity was anticipated across studies due to differences in diagnostic methods, geographic locations, study settings, and population characteristics. The random-effects model was therefore chosen to account for both within-study and between-study variability. Statistical heterogeneity among studies was assessed using Cochran’s Q test, with a significance level of p < 0.10 indicating the presence of heterogeneity, and quantified using the I² statistic. I² values were interpreted as low (25%), moderate (50%), and high (75%) heterogeneity. In addition, visual inspection of forest plots was used to further explore variability across studies. Subgroup analyses were conducted to investigate potential sources of heterogeneity, stratified by country, diagnostic method, and study setting. Where applicable, separate pooled prevalence estimates were calculated within each subgroup using the same random-effects approach. Publication bias was assessed through visual inspection of funnel plots to evaluate asymmetry. All statistical analyses were performed using STATA version 18 (StataCorp LLC, College Station, Texas, USA). | Page 6, 121-131 |
|  | 13e | To explore possible sources of heterogeneity among the included studies, subgroup analyses were conducted based on predefined study characteristics. These included geographical location (country), diagnostic method used for detection of Strongyloides stercoralis (microscopy-based techniques, concentration methods, and molecular or combined approaches), and study setting (community-based versus institution-based studies). Separate pooled prevalence estimates were calculated within each subgroup to examine variations in infection rates and identify potential contributors to between-study heterogeneity. In addition, visual inspection of forest plots was used to assess patterns of variability across studies within and between subgroups. Where sufficient data were available, differences in prevalence estimates were compared descriptively across categories to identify potential drivers of heterogeneity, particularly the influence of diagnostic sensitivity.  Meta-regression analysis was not performed due to limited number of studies within certain subgroups and variability in reporting of covariates across studies, which restricted the ability to conduct reliable multivariable exploration of heterogeneity. | Page 6, 121-131 |
|  | 13f | Sensitivity analyses were conducted to assess the robustness and stability of the pooled prevalence estimates. A leave-one-out sensitivity analysis was performed by sequentially excluding one study at a time from the meta-analysis to evaluate the influence of individual studies on the overall pooled prevalence of Strongyloides stercoralis infection among children. The consistency of pooled estimates after each exclusion was examined to determine whether any single study disproportionately affected the overall results. The sensitivity analyses were also carried out by excluding studies classified as low methodological quality based on the Joanna Briggs Institute (JBI) assessment tool. The pooled prevalence was then recalculated to determine whether study quality had a significant impact on the overall estimate. In addition, sensitivity analyses were also performed by comparing pooled estimates using different diagnostic categories to evaluate the effect of diagnostic approach on the robustness of the findings. Overall, the results remained consistent across these analyses, indicating that the findings were stable and not driven by a single study or specific | Page 6, 121-128 |
| Reporting bias assessment | 14 | Risk of bias due to missing results (reporting bias) was assessed using both graphical and qualitative approaches. Funnel plots were generated for the primary meta-analysis to visually inspect potential asymmetry, which may indicate publication bias or selective outcome reporting. Symmetry of the funnel plot was interpreted as an absence of major reporting bias, while asymmetry suggested possible small-study effects or selective publication of prevalence estimates. In addition, statistical assessment of publication bias was performed using standard tests for funnel plot asymmetry. The presence of missing studies or selective non-reporting was further evaluated by comparing study characteristics, sample sizes, and reported prevalence across included studies. A comprehensive search strategy, including multiple databases and grey literature sources, was employed to minimize the likelihood of missing relevant studies. No restrictions on publication year were applied, further reducing the risk of time-related publication bias. | Page 6, 129-131 |
| Certainty assessment | 15 | Observational studies were initially considered as low-certainty evidence and were then upgraded or downgraded depending on the presence and magnitude of limitations in the above domains. High heterogeneity and variability in diagnostic methods were key factors influencing the final certainty rating. No automated tools were used in this process. The assessment was conducted manually and independently by the reviewers, with disagreements resolved through discussion and consensus. | Page 6, line 131-32 |
| **RESULTS** | | |  |
| Study selection | 16a | The literature search yielded a total of 651 records from four electronic databases: Google Scholar (n = 334), PubMed (n = 189), Scopus (n = 102), and ScienceDirect (n = 26). After removal of duplicates, the remaining records were screened by title and abstract, followed by full-text assessment for eligibility based on predefined inclusion and exclusion criteria.  During the screening process, a substantial number of studies were excluded due to irrelevance to the study population (non-children or non-African settings), lack of outcome data on Strongyloides stercoralis prevalence, or inappropriate study design. After full-text review, studies that did not meet the eligibility criteria were excluded for reasons such as absence of usable prevalence data, focus on diagnostic method comparison only, or insufficient reporting of outcomes.  Ultimately, 27 studies met all inclusion criteria and were included in the qualitative and quantitative synthesis (meta-analysis). These studies collectively involved 16,358 children from eight African countries.  The entire selection process followed the Preferred Reporting Items for Systematic Reviews and Meta-Analyses (PRISMA Statement) guidelines and is summarized in a PRISMA flow diagram (Figure 1), which illustrates the stages of identification, screening, eligibility assessment, and final inclusion. | Page 7, line136-142 |
|  | 16b | During full-text screening, several studies that initially appeared relevant were excluded after detailed assessment against the eligibility criteria. The main reasons for exclusion included inappropriate population, lack of extractable prevalence data, non-eligible study design, or focus on methodological comparisons rather than epidemiological outcomes.  For example, some studies conducted in African countries reported Strongyloides stercoralis infections in mixed populations where results for children could not be separated from adults, and therefore these studies were excluded because age-specific data were not available. In other cases, studies primarily focused on clinical case series or hospital-based case reports that described infected individuals without providing denominator data (total number examined), making it impossible to calculate prevalence.  Additionally, certain studies were excluded because they evaluated diagnostic methods rather than reporting prevalence estimates, even though they included children in their sample. These studies did not provide population-based prevalence data required for meta-analysis. Studies that used only Kato-Katz technique as a single diagnostic tool for S. stercoralis detection were also excluded due to its known poor sensitivity for this parasite and inconsistency with the predefined diagnostic eligibility criteria.  Finally, duplicate publications reporting the same dataset in different formats or journals were identified and excluded to avoid double counting. In such cases, only the most complete or recent version of the study was retained for analysis.  Overall, exclusions were applied consistently according to the predefined criteria to ensure that only studies with comparable and extractable prevalence data among African children were included in the final synthesis. | Page 9-10, table 1 |
| Study characteristics | 17 | A total of 27 studies conducted between 2010 and 2024 were included in the systematic review and meta-analysis. These studies were conducted in eight African countries and included 16,358 children aged below 14 years. Most studies were cross-sectional in design and were conducted in school or community settings. Al articles are properly cited in the table 1 | Page 9-10, table 1 |
| Risk of bias in studies | 18 | Risk of bias was assessed for all 27 included studies using the Joanna Briggs Institute (JBI) critical appraisal checklist for prevalence studies. The assessment considered sampling methods, sample size adequacy, clarity of study population, validity of diagnostic methods, statistical analysis, and response rate. Each study was independently evaluated and categorized as having low (high risk of bias), moderate, or high quality (low risk of bias).  Overall, the methodological quality of the included studies varied. A considerable proportion of studies were assessed as moderate quality, largely due to limitations in sampling strategies (often convenience or school-based sampling), insufficient reporting of sample size justification, and reliance on low-sensitivity diagnostic techniques such as direct smear microscopy and formalin-ether concentration methods. These methodological limitations may have contributed to underestimation of the true prevalence of Strongyloides stercoralis infection.  Studies using more sensitive diagnostic approaches such as Baermann concentration, agar plate culture, or PCR generally demonstrated higher methodological quality scores due to improved diagnostic validity. However, even among these studies, some limitations were noted in reporting of sampling procedures and response rates.  No study was excluded based on risk of bias; all 27 studies were included in the quantitative synthesis. The distribution of risk of bias across studies was summarized narratively and is recommended to be presented in a tabular format (risk of bias table) in the manuscript or supplementary materials for clarity. |  |
| Results of individual studies | 19 | For each included study, data were extracted on the total number of children examined, the number of Strongyloides stercoralis–positive cases, and the corresponding prevalence estimates with their 95% confidence intervals. As this was a single-arm prevalence meta-analysis, no comparison groups were present; therefore, summary statistics consisted of sample size and number of infected individuals per study.  Across the 27 included studies, sample sizes ranged from 142 to 3,063 children, while the number of positive cases ranged from 1 to 329. Study-specific prevalence estimates varied widely, from as low as 0.07% in Bocanegra et al. conducted in Angola to as high as 48.5% in Aramendia et al. conducted in Ethiopia. Intermediate estimates included 27.1% in Côte d’Ivoire, 39.0% in Ethiopia, and 34.3% in Nigeria, reflecting substantial variability across study settings and diagnostic approaches.  Each study’s effect estimate was expressed as prevalence proportion, calculated as the number of infected children divided by the total sample size, and presented with 95% confidence intervals using binomial methods. These study-level estimates formed the basis for the pooled analysis using a random-effects model, which accounted for the observed heterogeneity across studies. Forest plots were used to visually present individual study estimates, their precision, and the overall pooled prevalence |  |
| Results of syntheses | 20a | For the main synthesis of pooled prevalence of Strongyloides stercoralis infection among African children, all 27 included studies contributed to the analysis. These studies were observational in design, predominantly cross-sectional, and conducted across eight African countries. Most studies were school-based, while a smaller proportion were community-based or health facility-based. There was substantial variability in sample sizes, ranging from small community surveys to large school-based studies. Diagnostic methods also varied considerably, with the majority of studies using low-sensitivity techniques such as direct smear microscopy and formalin-ether concentration, while a smaller proportion employed more sensitive methods such as Baermann concentration, agar plate culture, and PCR-based diagnostics.  In terms of risk of bias, most studies were assessed as having moderate methodological quality, with a smaller number rated as high quality. Common limitations included non-random or convenience sampling, inadequate reporting of sample size justification, and limited description of sampling procedures. A key source of bias across studies was the widespread use of low-sensitivity diagnostic methods, which likely contributed to underestimation of true infection prevalence. Despite these limitations, all studies were retained in the meta-analysis due to their relevance to the review question.  For subgroup syntheses, studies contributing to each category shared similar characteristics but still exhibited heterogeneity. For example, country-specific syntheses included studies with varying diagnostic approaches and settings, while diagnostic-method subgroups differed mainly in laboratory techniques used, with molecular and concentration-based methods generally showing higher methodological quality. Overall, risk of bias patterns were consistent across all syntheses, with diagnostic accuracy and sampling methodology being the main drivers of variability in study quality and prevalence estimates. |  |
|  | 20b | **Results of statistical syntheses**  A random-effects meta-analysis was conducted to estimate the pooled prevalence of *Strongyloides stercoralis* infection among African children. The overall pooled prevalence from the 27 included studies was 14.0% (95% CI: 12.2–15.9%). This indicates a considerable burden of infection among children across African settings.Substantial statistical heterogeneity was observed among the included studies, with Cochran’s Q = 2.20 × 10⁷, I² = 100%, and p ≤ 0.001, indicating extreme variability in prevalence estimates beyond that expected by chance. This high heterogeneity reflects differences in diagnostic methods, study populations, geographic regions, and study settings.  **Subgroup analyses**  Significant differences in pooled prevalence were observed across all predefined subgroups.   - **By country:** Prevalence varied significantly between countries (Q = 720,132.7, p < 0.001). The highest pooled prevalence was observed in Côte d’Ivoire (27%), followed by Ethiopia (26%) and Tanzania (17%). The lowest prevalence was reported in São Tomé and Príncipe (2%) and Ghana (3%). The direction of effect showed consistently higher prevalence in East and West African studies compared with West African low-burden settings. - **By diagnostic method**: There was a clear gradient in prevalence according to diagnostic sensitivity. Studies using combined molecular, culture, and microscopy methods showed the highest pooled prevalence (36%), followed by culture-based methods alone (23%), while microscopy-based methods (DSM/FEC) showed substantially lower prevalence estimates. This indicates a positive direction of effect, with more sensitive diagnostic methods yielding higher prevalence estimates. - **By study setting:** Significant differences were observed between settings (Q = 87.6, p < 0.001). School and clinic-based studies showed higher pooled prevalence (21%) compared with community-based studies (17%), indicating a modest increase in detected infection in institution-based settings.   **Summary of synthesis findings**  Overall, all statistical syntheses demonstrated high heterogeneity but consistent directional patterns: prevalence estimates increased with improved diagnostic sensitivity and varied significantly by geographic region and study setting. These findings highlight both the substantial burden of *S. stercoralis* infection among African children and the strong influence of diagnostic methodology on observed prevalence. |  |
|  | 20c | Investigations into the possible sources of heterogeneity among the included studies were conducted primarily through subgroup analyses based on predefined study characteristics, including country, diagnostic method, and study setting. These analyses demonstrated that heterogeneity was substantial and statistically significant across all subgroups (overall I² = 100%, p ≤ 0.001), indicating that variability in prevalence estimates was not attributable to chance alone.  Country-level subgroup analysis showed significant differences in pooled prevalence (Q = 720,132.7, p < 0.001), suggesting that geographic location was an important contributor to heterogeneity. Higher prevalence estimates were observed in East and West African countries, particularly in Ethiopia and Côte d’Ivoire, while markedly lower estimates were observed in Ghana and São Tomé and Príncipe. This indicates a strong geographical gradient in infection burden.  Diagnostic method was identified as another major source of heterogeneity. Studies using highly sensitive diagnostic approaches, such as PCR, Baermann concentration, and agar plate culture, reported substantially higher prevalence estimates (up to 36%) compared with those using conventional microscopy-based methods such as direct smear microscopy and formalin-ether concentration. This pattern demonstrates a clear methodological influence on heterogeneity, with more sensitive diagnostic tools yielding consistently higher detection rates.  Study setting also contributed to variability in prevalence estimates (Q = 87.6, p < 0.001). Institution-based studies (school and clinic settings) reported higher pooled prevalence (21%) compared with community-based studies (17%), suggesting that differences in sampling environments and population characteristics partially explained between-study heterogeneity.  Overall, these findings indicate that heterogeneity in the pooled prevalence estimates was mainly driven by differences in diagnostic sensitivity, geographic variation, and study setting, rather than random variation alone. No additional meta-regression analyses were performed due to limitations in the number of studies within certain subgroups and incomplete reporting of covariates across studies. |  |
|  | 20d | Sensitivity analyses were performed to assess the robustness and stability of the pooled prevalence estimate of Strongyloides stercoralis infection among African children. A leave-one-out sensitivity analysis was conducted by sequentially omitting each study from the meta-analysis to evaluate the influence of individual studies on the overall pooled estimate. The results showed that exclusion of any single study did not substantially alter the pooled prevalence, which remained stable around 14%, indicating that no single study had a disproportionate effect on the overall estimate.  Additional sensitivity analyses were performed by excluding studies classified as low methodological quality based on the Joanna Briggs Institute (JBI) appraisal tool. The pooled prevalence after exclusion of low-quality studies remained consistent with the main analysis, with only minor changes in magnitude and no change in overall interpretation, confirming the robustness of the findings.  Further sensitivity analysis was conducted by restricting the analysis to studies that used more sensitive diagnostic methods (Baermann concentration, agar plate culture, PCR, or combined techniques). Although this restriction resulted in a higher pooled prevalence estimate compared with the overall analysis, the direction of effect and pattern of findings remained consistent, supporting the influence of diagnostic sensitivity but not altering the overall conclusion.  Overall, the sensitivity analyses demonstrated that the pooled prevalence estimate was robust and not driven by any single study, methodological quality category, or diagnostic subgroup. These findings strengthen the reliability of the main meta-analytic results. |  |
| Reporting biases | 21 | Risk of bias due to missing results (reporting bias) was assessed for the main synthesis of pooled prevalence of Strongyloides stercoralis infection among African children, as well as for subgroup analyses by country, diagnostic method, and study setting.  For the overall meta-analysis, visual inspection of the funnel plot suggested a generally symmetrical distribution of studies around the pooled effect estimate, indicating no strong evidence of publication bias or small-study effects. This pattern suggested a low risk of bias due to missing studies in the primary synthesis, although some minor asymmetry among smaller studies could not be fully excluded.  For subgroup syntheses, the assessment of reporting bias was limited by the reduced number of studies within certain strata, particularly country-specific and diagnostic-method subgroups. In these cases, funnel plot interpretation was less reliable; however, no clear or consistent asymmetry was observed across subgroup analyses. This suggests that the risk of reporting bias was not substantial enough to meaningfully distort subgroup-specific pooled estimates.  Across all syntheses, a comprehensive search strategy involving multiple databases and grey literature sources was used to minimize the likelihood of missing studies. No restrictions on publication year were applied, further reducing the potential for time-lag bias. Nevertheless, the possibility of unpublished studies or selective non-reporting of low-prevalence findings cannot be completely excluded, particularly given the reliance on published observational studies in resource-limited settings.  Overall, while minor uncertainty remains due to the inherent limitations of observational prevalence studies, the available evidence did not indicate substantial risk of bias arising from missing results across any of the conducted syntheses. |  |
| Certainty of evidence | 22 | The certainty (confidence) in the body of evidence for the main outcome—pooled prevalence of *Strongyloides stercoralis* infection among African children—was assessed using the Grading of Recommendations Assessment, Development and Evaluation (GRADE) approach. Because all included studies were observational in design, the initial level of certainty was considered low and was then evaluated across the domains of risk of bias, inconsistency, indirectness, imprecision, and publication bias.  Overall, the certainty of evidence for the primary outcome was rated as low to moderate. The certainty was downgraded primarily due to very high statistical heterogeneity (I² = 100%), reflecting substantial inconsistency in prevalence estimates across studies. Additional downgrading was applied due to methodological limitations, particularly the frequent use of low-sensitivity diagnostic techniques and non-random sampling approaches in many studies. However, the large cumulative sample size (16,358 children) and inclusion of multiple African countries contributed to strengthening the evidence base.  For subgroup outcomes (country-specific prevalence, diagnostic-method-specific prevalence, and study-setting-specific prevalence), the certainty of evidence was generally rated as low. This was due to smaller numbers of studies within subgroups, wide variability in prevalence estimates, and persistent heterogeneity within each category. Despite these limitations, consistent directional patterns were observed, particularly the higher prevalence associated with more sensitive diagnostic methods and certain geographic regions.  No evidence was found to warrant upgrading the certainty of evidence. Although publication bias was not strongly indicated by funnel plot assessment, the possibility of undetected or unpublished studies could not be fully excluded, contributing to further caution in interpretation.  Overall, the body of evidence supporting the pooled prevalence of *S. stercoralis* infection among African children was considered of low certainty, indicating that the true prevalence may differ from the estimated pooled value, but the direction of findings (substantial burden and diagnostic influence) is likely reliable. |  |
| **DISCUSSION** | | |  |
| Discussion | 23a | Overall, the findings of this systematic review and meta-analysis indicate that Strongyloides stercoralis infection remains an important but underrecognized parasitic infection among children in Africa, with a pooled prevalence of about 14%. This estimate is higher than many individual country reports in the literature, suggesting that the regional burden is likely underestimated when only low-sensitivity diagnostic approaches are used.  When compared with global evidence, the results are consistent with previous systematic reviews from other tropical and subtropical regions, which have also demonstrated that prevalence estimates vary widely depending on diagnostic methods and study populations. Studies from Southeast Asia and parts of Latin America have similarly reported markedly higher prevalence when using sensitive techniques such as agar plate culture, Baermann concentration, or PCR, compared with conventional microscopy. This aligns with the present findings, where diagnostic method emerged as a major determinant of observed prevalence, with molecular and concentration-based methods yielding substantially higher detection rates than direct smear microscopy or formalin-ether concentration.  The observed heterogeneity across African countries is also consistent with previous epidemiological evidence indicating that S. stercoralis distribution is highly focal and influenced by environmental, socioeconomic, and behavioral factors such as sanitation, poverty, and soil exposure. The higher prevalence observed in countries such as Ethiopia and Côte d’Ivoire mirrors findings from other helminth-endemic settings where ecological conditions and diagnostic intensity are greater.  Importantly, the relatively lower prevalence reported in some studies does not necessarily indicate a lower true burden, but rather reflects methodological limitations, particularly reliance on low-sensitivity diagnostic techniques. This interpretation is supported by global consensus that conventional stool microscopy significantly underestimates S. stercoralis infections due to intermittent larval shedding and low parasite burden.  In comparison with global estimates suggesting hundreds of millions of infections worldwide, the present findings reinforce the view that Africa contributes substantially to the global burden, although the true magnitude remains uncertain. The consistency of higher prevalence with improved diagnostic methods across studies strengthens the evidence that diagnostic sensitivity is the key driver of underestimation.  In summary, the results of this review align with and extend existing global evidence by confirming that S. stercoralis infection among African children is both widespread and systematically underestimated. This underscores the need for improved diagnostic strategies and more robust epidemiological surveillance to better define its true public health impact. | Page 14, line 180-6 |
|  | 23b | The evidence included in this systematic review has several important limitations that should be considered when interpreting the findings. First, most of the included studies were cross-sectional in design, which limits the ability to infer temporal patterns of infection or causal relationships between risk factors and Strongyloides stercoralis infection. Second, Methodological inconsistency reduces comparability across studies and limits the precision of the pooled estimate. Third, some studies had relatively small sample sizes These reporting deficiencies contributed to moderate methodological quality in many studies and may have introduced risk of bias in prevalence estimates although comprehensive searching has been done. | Page 16, line 213-15 |
|  | 23c | Although a comprehensive search strategy was applied across multiple databases (PubMed, Scopus, ScienceDirect, and Google Scholar), the search was restricted to studies published in English. Despite the inclusion of multiple databases and manual reference screening, the possibility of missing unpublished studies or grey literature cannot be fully excluded. Although study selection and data extraction were performed independently by two reviewers with consensus procedures, no automation tools or machine learning-assisted screening methods were used. While manual screening enhances careful judgment, it is time-consuming and may still be subject to human error or subjective interpretation, particularly during title and abstract screening. | Page 16, line 215-15 |
|  | 23d | The findings of this systematic review and meta-analysis have important implications for clinical practice, public health policy, and future research on *Strongyloides stercoralis* infection among African children.  From a clinical and diagnostic practice perspective, the results highlight the substantial underestimation of infection when relying on conventional microscopy-based techniques such as direct smear microscopy and formalin-ether concentration. The markedly higher prevalence observed in studies using Baermann concentration, agar plate culture, and molecular diagnostics indicates that these more sensitive methods should be prioritized where feasible. Clinicians and laboratory personnel in endemic settings should be aware of the limitations of routine diagnostic methods and consider integrating more sensitive techniques, especially in high-risk pediatric populations.  In terms of public health policy, the findings underscore that S. stercoralis remains a neglected component of soil-transmitted helminth control programs in Africa. Policymakers should consider integrating strongyloidiasis into existing neglected tropical disease (NTD) control frameworks, including school-based deworming programs and national surveillance systems. For future research, there is a clear need for well-designed epidemiological studies using standardized, high-sensitivity diagnostic protocols to generate more accurate burden estimates. | Page 14-16, line 184-212 |
| **OTHER INFORMATION** | | |  |
| Registration and protocol | 24a | The protocol for this systematic review and meta-analysis was prospectively registered in the International Prospective Register of Systematic Reviews (PROSPERO). The assigned registration number is **CRD420261325085**.This registration ensured transparency of the review methods and helped minimize the risk of duplication and selective reporting of outcomes |  |
|  | 24b | The review protocol was prospectively registered in the International Prospective Register of Systematic Reviews (PROSPERO) and can be accessed online through the PROSPERO database using the registration number **CRD420261325085**. The full protocol is available on the PROSPERO website and contains the predefined objectives, eligibility criteria, and methodological approach used for this systematic review and meta-analysis. |  |
|  | 24c | Several minor amendments were made to the original registered protocol during the conduct of the review to improve clarity and accommodate available evidence, while maintaining the overall objectives and methodological framework. For instance, additional electronic databases (such as ScienceDirect and Google Scholar) were included beyond the initially planned sources to ensure a more comprehensive search and to capture relevant grey literature. The diagnostic method classification was also refined during data extraction. |  |
| Support | 25 | The systematic review and meta-analysis did not receive any specific financial support from public, commercial, or not-for-profit funding agencies. No external funding body was involved in the design, data collection, analysis, interpretation, or writing of the review. All activities related to the review, including literature search, data extraction, statistical analysis, and manuscript preparation, were conducted independently by the authors as part of their academic and research responsibilities. |  |
| Competing interests | 26 | The authors declare that they have no competing interests. All authors confirm that they have no financial, personal, or professional relationships that could inappropriately influence or bias the conduct, interpretation, or reporting of this systematic review and meta-analysis. |  |
| Availability of data, code and other materials | 27 | Data will available freely online at Bahir Dar University:  The datasets generated and/or analyzed during the current study are available from the corresponding author upon reasonable request |  |

*From:* Page MJ, McKenzie JE, Bossuyt PM, Boutron I, Hoffmann TC, Mulrow CD, et al. The PRISMA 2020 statement: an updated guideline for reporting systematic reviews. BMJ 2021;372:n71. doi: 10.1136/bmj.n71. This work is licensed under CC BY 4.0. To view a copy of this license, visit <https://creativecommons.org/licenses/by/4.0/>
