# Supplementary material for: Prevalence of Strongyloides stercoralis infection among African children: A systematic review and meta-analysis
Source: PLoS Negl Trop Dis. 2026 Oct 7;20(10):e0014759. doi: 10.1371/journal.pntd.0014759 (PMC13667923; doi:10.1371/journal.pntd.0014759)
Supplement: Supplementary file 3 [file pntd.0014759.s004.docx]

**Authors’ response to editor’s and reviewers’ comments**

We sincerely thank the editor and the reviewers for their thoughtful, constructive, and insightful comments, which have significantly improved the quality and clarity of our manuscript. We have carefully considered every comment and revised the manuscript accordingly. A detailed point-by-point response has been provided for each comment, and all corresponding changes have been incorporated into the revised manuscript and highlighted in yellow for ease of reference. We hope that our revisions have adequately addressed all of the editor's and reviewers' concerns. If any further revisions is required, we would be grateful for the opportunity to provide them and further improve the manuscript.

**Editor’s comment**

If the reviewer comments include a recommendation to cite specific previously published works, please review and evaluate these publications to determine whether they are relevant and should be cited. There is no requirement to cite these works unless the editor has indicated otherwise.

1) We noticed that you used the phrase 'unpublished data' in the manuscript. We do not allow these references, as the PLOS data access policy requires that all data be either published with the manuscript or made available in a publicly accessible database. Please amend the supplementary material to include the referenced data or remove the references.

**Authors’ response:** Thank you for bringing this to our attention. We have removed all references to "unpublished data" from the manuscript and supplementary materials.

2) Please upload all main figures as separate Figure files in .tif or .eps format. For more information about how to convert and format your figure files please see our guidelines:
   <https://journals.plos.org/plosntds/s/figures>

**Authors’ response**: Thank you for your guidance. We have converted all main figures into separate figure files in **.tif format** according to the journal’s figure preparation guidelines and uploaded them as individual figure files during the resubmission process. We appreciate your assistance and consideration.

3) Please upload a copy of Figure Fig 1-4 which you refer to in your text on pages 4, 7, 12, and 14. Or, if the figure is no longer to be included as part of the submission please remove all reference to it within the text.

**Authors’ response:** Thank you for your comment. We confirm that **Figures 1–4 are still included as part of the manuscript** and have been uploaded as separate figure files during the resubmission process. The corresponding references to these figures within the main text have been retained and checked to ensure consistency with the uploaded figure files. We appreciate your careful review and guidance.

4) We have noticed that you have uploaded Supporting Information files, but you have not included a list of legends. Please add a full list of legends for your Supporting Information files after the references list.

**Authors’ response:** Thank you for pointing this out. We have added a complete list of legends for all Supporting Information files after the References section, as requested. The legends have been checked to ensure that they accurately correspond to the respective Supporting Information files. We appreciate your helpful guidance. (line 383)

5) As required by our policy on Data Availability, please ensure your manuscript or supplementary information includes the following:

- A numbered table of all studies identified in the literature search, including those that were excluded from the analyses.

- For every excluded study, the table should list the reason(s) for exclusion.
- If any of the included studies are unpublished, include a link (URL) to the primary source or detailed information about how the content can be accessed.

- A table of all data extracted from the primary research sources for the systematic review and/or meta-analysis. The table must include the following information for each study:
 -Name of data extractors and date of data extraction

-Confirmation that the study was eligible to be included in the review.

- All data extracted from each study for the reported systematic review and/or meta-analysis that would be needed to replicate your analyses.

- If data or supporting information were obtained from another source (e.g. correspondence with the author of the original research article), please provide the source of data and dates on which the data/information were obtained by your research group.

- If applicable for your analysis, a table showing the completed risk of bias and quality/certainty assessments for each study or outcome.  Please ensure this is provided for each domain or parameter assessed. For example, if you used the Cochrane risk-of-bias tool for randomized trials, provide answers to each of the signalling questions for each study. If you used GRADE to assess certainty of evidence, provide judgements about each of the quality of evidence factor. This should be provided for each outcome.

-An explanation of how missing data were handled.

This information can be included in the main text, supplementary information, or relevant data repository. Please note that providing these underlying data is a requirement for publication in this journal, and if these data are not provided your manuscript might be rejected.

**Authors’ response:** Thank you for your important comment regarding the Data Availability requirements. We have carefully revised the manuscript and supplementary materials to ensure full compliance with the journal’s Data Availability policy. Specifically, we have added a complete list of all studies identified through the literature search, including both included and excluded studies, with the reasons for exclusion provided for each excluded study All relevant supplementary files have been uploaded with the revised submission, and the manuscript has been updated to clearly indicate where these supporting data can be accessed. We appreciate the editor's guidance, which has helped us improve the transparency, completeness, and reproducibility of our systematic review and meta-analysis (Supplementary Table S1).

6) We have amended your Competing Interest statement to comply with journal style. We kindly ask that you double check the statement and let us know if anything is incorrect.

**Authors’ response:** Thank you for your careful review and for amending the Competing Interest statement to comply with the journal’s style requirements. We have checked the revised statement and confirm that it is accurate and reflects the authors’ declarations appropriately. No further corrections are required.

**Reviewers' Comments:**

**Key Review Criteria Required for Acceptance.**

**Methods**
-Are the objectives of the study clearly articulated with a clear testable hypothesis stated?
-Is the study design appropriate to address the stated objectives?

-Is the population clearly described and appropriate for the hypothesis being tested?
-Is the sample size sufficient to ensure adequate power to address the hypothesis being tested?
-Were correct statistical analysis used to support conclusions?

-Are there concerns about ethical or regulatory requirements being met?

**Reviewer #1**:

-Are the objectives of the study clearly articulated with a clear testable hypothesis stated? No

**Authors’ response:** Thank you for this valuable comment. We have revised the Introduction to clearly articulate the hypothesis and study objectives. These revisions improve the clarity of the study aims and ensure that the objectives and hypothesis are well aligned with the introduction, methodology and outcomes of the systematic review and meta-analysis. We appreciate the reviewer’s suggestion, which has strengthened the manuscript (line 47-56).

-Is the study design appropriate to address the stated objectives? Yes

**Authors’ response:** Thank you for your positive assessment.

-Is the population clearly described and appropriate for the hypothesis being tested? No

**Authors’ response:** Thank you for your comments. Revision has been done in the revised manuscript. The study population is the African children (Line 52-53)

-Is the sample size sufficient to ensure adequate power to address the hypothesis being tested? Yes

**Authors’ response:** Thank you for your positive assessment.

-Were correct statistical analysis used to support conclusions? Yes

**Authors’ response:** Thank you for your positive assessment and for acknowledging that the statistical analyses appropriately support our study conclusions.

-Are there concerns about ethical or regulatory requirements being met? No

**Authors’ response:** Thank you for your positive assessment. We appreciate the reviewer’s confirmation that there are no concerns regarding compliance with ethical or regulatory requirements

**Reviewer #2:**

The methods are described clearly in the M&M section, however, there are some issues. Here are some things to take into consideration:

1. On Figure 1 “Overview of search methods articles inclusion and exclusion criteria”. The right-hand side panel, where “Identification of studies via other methods is mentioned” does not add anything to the manuscript, especially since no studies were selected for assessment in this way. Please use the appropriate PRISMA Flow Diagram for this review.

**Authors’ response:** Thank you for this valuable comment. We agree that no studies were identified through additional methods such as citation searching or other sources, and therefore the “Identification of studies via other methods” pathway was not applicable to our review. However, we retained the original PRISMA 2020 flow diagram format because it was prepared according to the journal’s pre-review editorial requirements. The relevant section has been reported as having no identified studies, and we have clarified this point to avoid any potential confusion.

2. Still on Figure 1, what was the total number of records (651?) that were retrieved from the 4 databases, from which 100 were duplicates, 39 records marked as ineligible by automation tools and the 3 removed for other reasons?

**Authors’ response:** Thank you for this comment. Yes, the total number of records retrieved from the four electronic databases was 651, as indicated in the Results section of the manuscript (line 137) and we included in Fig 1. Of these, 100 duplicate records, 39 records identified as ineligible by automation tools, and 3 records removed for other reasons were excluded before screening. We have ensured that the numbers presented in Figure 1 are consistent with the information provided in the Results section.

3. Please describe in detail within the Materials and Methods which automation tools were used before the initial screening of records. Questions arising from this include: Was this an artificial intelligence tool? What were the criteria/thresholds for these tools to decide which records to keep or not? And were the records excluded by these tools reviewed to ensure relevant studies were not mistakenly removed?

**Authors’ response:** Thank you for this important comment. We apologize for the confusion caused by the incorrect information in the PRISMA flow diagram. No automation tools or artificial intelligence-assisted screening methods were used during the study selection process. The statement "Records marked as ineligible by automation tools (n = 38)" was included in error and has been corrected in the revised Figure 1. The 38 records were manually screened based on their titles and abstracts according to the predefined eligibility criteria and were excluded because they did not meet the inclusion criteria. We have revised the Materials and Methods section accordingly to accurately describe the manual screening process and ensure consistency throughout the manuscript.4. Still on Figure 1, there are some asterisk markings (“*”), which I presume would indicate the need for footnotes or something similar, which are not presented within the figure itself or on its caption.

**Authors’ response:** Thank you for this careful observation. We agree that the asterisk markings (“*”) in Figure 1 were unclear because they were not accompanied by corresponding footnotes in the figure or legend. These symbols were inadvertently included during preparation of the PRISMA flow diagram and did not represent any additional information or specific notes. We have therefore removed the unnecessary asterisk markings from Figure 1 to improve clarity and avoid confusion.

5. Figure 1 in general, needs to be revised and improved very carefully. There are stages where it just states “Records excluded” without providing the reason, or even “Records screened (n=510)” without stating what was used to screen (e.g., title and abstract, etc.).

**Authors’ response:** Thank you for this important comment. We agree that the original Figure 1 did not provide sufficient detail regarding the screening process and reasons for exclusion. We have carefully revised the PRISMA flow diagram to improve clarity and transparency. We have provided the total retrieved (n=651), remove the ‘*’. The revised Figure 1 now more accurately reflects the study identification, screening, eligibility assessment, and inclusion processes described in the Materials and Methods section.

Reviewer #3: The study has a clear and appropriate study design. The methods used to conduct the systematic review and meta-analysis are appropriate and adequate.

**Authors’ response:** Thank you very much for your positive evaluation of our study design and methodology. We appreciate your recognition that the methods used for conducting the systematic review and meta-analysis were appropriate and adequate. We have carefully considered all reviewer comments and further improved the manuscript to enhance clarity, transparency, and methodological rigor.

**Results**

-Does the analysis presented match the analysis plan?

-Are the results clearly and completely presented?

-Are the figures (Tables, Images) of sufficient quality for clarity?

Reviewer #1:

- Does the analysis presented match the analysis plan? There are issues with conducting the analyses.

**Authors’ response:** Thank you for this important comment. We appreciate the reviewer’s careful assessment of the analytical approach. We have re-examined the analyses presented in the manuscript in relation to the predefined analysis plan to ensure consistency between the planned methods and the reported results

-Are the results clearly and completely presented? The results are well presented, but they have some problems.

**Authors’ response:** Thank you for this constructive comment. We appreciate the reviewer’s recognition that the results are generally well presented. We have carefully reviewed the Results section and revised the relevant parts to address the identified issues, improve clarity, and ensure that the findings are presented more completely and accurately.

-Are the figures (Tables, Images) of sufficient quality for clarity? Yes

**Authors’ response:** Thank you for your positive assessment. We appreciate the reviewer’s confirmation that the figures, tables, and images are of sufficient quality and clarity. We have carefully checked the presentation of all figures and tables in the revised manuscript.

Reviewer #2:

1. Line 144 and Table 1: please include the meaning of each and every abbreviation used in Table, since these are included only for DSM, FEC, BCT and APC, but are missing for qPCR, PCR, RT-PCR, STST. Also, please review the way the names of countries in the table are spelled to correct typos: “Co^te d’Ivory” should either be “Côte d’Ivoire” or “Ivory Coast”.

**Authors’ response:** Thank you for this helpful comment. We have been defined all abbreviations used in Table 1 (line 110-111). We have also carefully reviewed the names of countries presented in Table 1 and corrected typographical errors. Specifically, “Co^te d’Ivory” has been corrected to “Côte d’Ivoire”

2. Please be careful and consistent with the abbreviation for real-time Polymerase Chain Reaction. In some instances “qPCR” is used, and in others, “RT-PCR”. However, the latter can be confused with reverse-transcriptase Polymerase chain reaction, which is a different method. Please verify this throughout the entire manuscript.

**Authors’ response:** Thank you for this important observation. We agree that the inconsistent use of the abbreviations could cause confusion, as “RT-PCR” may be interpreted as reverse transcription polymerase chain reaction rather than real-time PCR. We have carefully reviewed the entire manuscript and standardized the terminology. All instances referring to real-time quantitative amplification have been corrected to **qPCR (quantitative polymerase chain reaction) in the result section**.

Reviewer #3:

Most of the results are clearly presented. However, there is a discrepancy in some interpretations between the results and the discussion sections. The discrepancies are stated in the attached report. There are a few grammatical errors as well.

**Authors’ response:** Thank you for this valuable comment. We appreciate the reviewer’s careful assessment of the manuscript. We have thoroughly reviewed the Results and Discussion sections to identify and correct inconsistencies in the interpretation of findings. The discrepancies highlighted in the attached report have been carefully addressed, and the relevant sections have been revised to ensure that the interpretations presented in the discussion accurately reflect the results. In addition, we have carefully edited the manuscript to correct grammatical errors and improve overall clarity and readability.

**Summary of the research**

The manuscript addresses a critical public health gap by pooling data on the prevalence of *Strongyloides stercoralis* among African children up to 2025. The study appropriately highlights that widespread reliance on low-sensitivity diagnostic tools (DSM, FEC) significantly underestimates the true disease burden. The systematic integration of 27 studies comprising 16,358 children provides a valuable epidemiological baseline. However, the text requires substantial structural, statistical, and grammatical refinements before it is suitable for publication.

**Authors’ response:** Thank you very much for the comprehensive evaluation of our manuscript. We appreciate the reviewer’s recognition of the importance of addressing the burden of Strongyloides stercoralis infection. In response to the reviewer’s suggestions, we have carefully revised the manuscript to improve the overall structure, clarify the statistical methods and results, strengthen the interpretation of findings, and correct grammatical and typographical errors throughout the text.

**Minor Revisions and Methodological Issues**

1. **Discrepancy in Data and Text Presenta0on in Settings Findings**: In the Result section, the text states: "The highest prevalence was reported among samples collected in school and clinic settings (21%), followed by community-based samples (17%)." However, in the Discussion section, the text contradicts this: "There was also observed a variation in the study results between different sampling settings, with higher prevalence observed in the community compared to school-based studies." Resolve this contradiction to ensure data integrity.

**Authors’ response**: Thank you for identifying this inconsistency. We agree that the interpretation of prevalence by sampling setting required clarification. Although the pooled prevalence was higher among samples from school and clinic settings (21%), this estimate was based on only one study and may not be representative in the discussion part. In contrast, the community-based estimate was derived from a larger number of studies, providing a more reliable basis for comparison. To avoid misleading interpretation, we have revised the Results and Discussion sections. In the revised manuscript, we emphasize the comparison between community-based studies and school-based studies, where sufficient numbers of studies were available for interpretation. We have also clarified that the higher prevalence observed in the single school/clinic-based study should be interpreted cautiously due to the limited evidence base. These revisions ensure that the conclusions are aligned with the available data and avoid over interpretation of findings from a single study.

2. **Clarification of Acronyms and Diagnostic Subgroups**
i. Undefined Acronyms: Even though the diagnostic methods introduced as acronyms were defined in Table 1, there should be prior definitions upon first mention for BCT, APC, FEC, and DSM (e.g., Formol-Ether Concentra0on, Direct Stool Microscopy) in the text.
ii. **Authors’ response**: Thank you for this comment. We agree that all acronyms should be clearly defined at their first appearance in the manuscript. However, the abbreviations for the diagnostic methods, including BCT, APC, FEC, and DSM, were already defined in the Introduction section at their first mention. We have nevertheless reviewed the manuscript carefully to ensure that all diagnostic method abbreviations are consistently defined at first use and are clearly presented in both the main text and Table 1. (Line 47-51)
iii. Subgroup Definition: Labelling methods as "better sensitive diagnostic methods" is subjective. Clearly specify what qualifies as "advanced/high-sensitivity" (such as molecular PCR, agar plate culture) versus "traditional/low-sensitivity" in both the methodology and results sections.
iv. **Authors’ response**: Thank you for this valuable comment. We agree that the term "better sensitive diagnostic methods" is subjective and may be unclear. Accordingly, we have revised the manuscript to use the more objective terms "high-sensitivity diagnostic methods" and "low-sensitivity diagnostic methods" throughout the Methods (line. 50, 113 &147)
3. **Interpretation of Extreme Heterogeneity**
i. The meta-analysis shows an I² of 100% (Q = 2.20e+07, P ≤ 0.001). An I² at this level signifies complete heterogeneity, suggesting that a single pooled prevalence estimate of 14.0% might not be clinically useful.
ii. **Authors’ response**: Thank you for this important comment. We agree that the observed heterogeneity was extremely high (I² = 100%), indicating substantial variability among the included studies and that the pooled prevalence estimate should be interpreted with caution. The high heterogeneity is likely related to differences among studies in geographical location, study population characteristics, sampling settings, diagnostic methods, and other methodological factors. To address this concern, we have revised the manuscript to provide a more cautious interpretation of the pooled prevalence estimate and have emphasized that it represents an overall summary measure rather than a uniform estimate applicable to all settings. We have also expanded the discussion of potential sources of heterogeneity and clarified the limitations associated with pooling prevalence estimates under conditions of extreme heterogeneity. Furthermore, subgroup analyses based on relevant study characteristics (e.g., diagnostic methods and sampling settings) were considered/presented to explore possible contributors to the observed variability
iii. The Discussion section should expand on this limitation. Emphasize that the pooled figure is a mathematical average across highly diverse study designs, geographic features, and diagnostic sensitivities, rather than a uniform representation of the continent.

**Authors’ response**: Thank you for this valuable suggestion. We agree that the interpretation of the pooled prevalence estimate requires greater emphasis regarding the influence of heterogeneity among the included studies. We have revised the discussion section to clarify that the pooled estimate represents a statistical summary across studies with substantial differences in study designs, geographical settings, population characteristics, sampling approaches, and diagnostic methods with varying sensitivities. Therefore, it should not be interpreted as a uniform estimate of S. stercoralis prevalence across the African continent, but rather as an overall indication of the burden reported in the available literature. We have also highlighted this limitation and emphasized the need for context-specific epidemiological studies using sensitive diagnostic approaches (line 184-89).

4. **Corrections for grammatical errors and syntax issues**
i. Third-Person Reference Issue: In the Discussion section (paragraph 1), the text states: "The authors clearly demonstrated that..." Since this is the authors' own manuscript, rephrase this to avoid writing in the third person about your own work (e.g., "This review demonstrates that..." or "Our findings demonstrate that...").

**Authors’ response**: Thank you for this helpful comment. We agree that referring to ourselves in the third person was inappropriate. We have revised the statement as recommended. (Line 144)

ii. Awkward phrasing: Current: "In the 27 studies, 16,358 children who were younger than 14 years old were included."

Correction: "The 27 included studies comprised 16,358 children under 14 years of age."

Current: "There was also observed a variation..." Correction: "Variation was also observed..."

**Authors’ response**: Thank you for this helpful comment. We agree that these sentences required improvement for clarity, conciseness, and readability. We have revised the wording throughout the manuscript accordingly. Specifically, the sentence “In the 27 studies, 16,358 children who were younger than 14 years old were included” has been revised to “The 27 included studies comprised 16,358 children under 14 years of age.” In addition, “There was also observed a variation...” has been revised to “Variation was also observed...” These changes improve the overall flow and readability of the manuscript.(line: 105, line:170)

**Recommendation to the Editor Decision:** Minor Revision

The manuscript has significant epidemiological value and focuses on a neglected tropical disease. However, it cannot be accepted until the contradictions between the Results and Discussion sections are clearly resolved and the manuscript has been carefully copy-edited.

**Authors’ response**: Thank you for your constructive evaluation and recommendation for minor revision. We appreciate the reviewer’s recognition of the epidemiological importance of our study and its contribution to understanding the burden of S. stercoralis infection among African children

The authors did not acknowledge the limitations of their review, noting that the studies varied in their designs, geographic regions, and diagnostic methods.

**Authors’ response:** Thank you for this important comment. We agree that the limitations of the review should be more explicitly acknowledged. Accordingly, we have revised the Discussion section to include a dedicated limitations paragraph. (Line 184-89)

**Conclusions**

-Are the conclusions supported by the data presented?

-Are the limitations of analysis clearly described?

-Do the authors discuss how these data can be helpful to advance our understanding of the topic under study?

-Is public health relevance addressed?

**Reviewer #1:** -Are the conclusions supported by the data presented? Yes

**Authors’ response:** Thank you for your positive assessment. We appreciate the reviewer’s confirmation that the conclusions are supported by the data presented.

-Are the limitations of analysis clearly described? No

**Authors’ response:** Thank you for this important comment. We agree that the limitations of the analysis were not sufficiently described in the original manuscript. These revisions provide a more balanced interpretation of the findings and their generalizability. (Line 184-89)

-Do the authors discuss how these data can be helpful to advance our understanding of the topic under study? No

**Authors’ response:** Thank you for this valuable comment. We agree that the original manuscript did not sufficiently discuss how the findings advance current understanding of the topic. We have revised to emphasize the contribution of this review in providing an updated estimate of the burden of S. stercoralis infection among African children, highlighting the impact of diagnostic methods on prevalence estimates, identifying important evidence gaps, and informing future research, surveillance, and control strategies in the recommendation section (196-99).

-Is public health relevance addressed? Yes!

**Authors’ response:** Thank you for your positive assessment. We appreciate the reviewer’s confirmation

Reviewer #2: Yes, the manuscript’s conclusions are mostly supported by the data presented, and limitations are also described, however, there are some issues in both the discussion and conclusions.
1. In the last paragraph of the Discussion (lines 215-217), the authors mention that “Additional limitations included restriction to English-language publications, the possibility of missing unpublished studies or grey literature, and the absence of automation tools or machine learning-assisted screening methods during the study selection process”. However, as mentioned in earlier comments, in Figure 1, authors have indicated that 38 records were excluded from the analysis as “Records marked as ineligible by automation tools (n = 38)”. Please clarify whether automation tools were used or not, and describe that appropriately in the Materials and Methods section of the manuscript.

**Authors’ response:** Thank you for this important observation. We apologize for the inconsistency between the Discussion and Figure 1. No automation tools or artificial intelligence-assisted screening methods were used during the study selection process. The statement "Records marked as ineligible by automation tools (n = 38)" in the PRISMA flow diagram was included inadvertently and has been corrected in the revised Figure 1. The Materials and Methods section has also been revised to clarify that all records were screened manually based on their titles and abstracts using the predefined eligibility criteria (line 71). The statement referring to the absence of automation tools has been considered in the limitations section (line 184-89)

2. The conclusion states (lines 224-226) that “Thus, we urge researchers to strive towards establishing standard procedures for diagnosing strongyloidiasis and write policy briefs that promote the inclusion of S. stercoralis in the soil-transmitted helminths control and prevention package.”. However, the “WHO guideline on preventive chemotherapy for public health control of strongyloidiasis” in July 2024; therefore, authors should take this into consideration when making such a statement.

**Authors’ response: T**hank you for this important comment. We appreciate the reviewer for highlighting the WHO Guideline on Preventive Chemotherapy for Public Health Control of Strongyloidiasis (2024). We have revised the conclusion accordingly to acknowledge this important development and modified the recommendation to emphasize the implementation of the WHO guideline, while highlighting the continued need for researchers to strive towards establishing standard procedures for diagnosing strongyloidiasis and generating epidemiological evidence to support surveillance and control efforts in endemic settings. (line 185-88)

Reviewer #3:

The conclusion represents the findings in the manuscript. The authors provided an important policy recommendation of including S.stercoralis infection in the soil-transmitted helminths control and prevention package.

**Authors’ response:** Thank you very much for your positive assessment. We appreciate the reviewer’s recognition that the conclusion accurately reflects the findings of the manuscript.

**Editorial and Data Presentation Modifications?**

Use this section for editorial suggestions as well as relatively minor modifications of existing data that would enhance clarity. If the only modifications needed are minor and/or editorial, you may wish to recommend “Minor Revision” or “Accept”.

Reviewer #1: The authors should revise the English.

**Authors’ response:** Thank you for this suggestion. We agree that improving the language quality is important for clarity and readability. The manuscript has been carefully reviewed and thoroughly edited to correct grammatical errors, improve sentence structure, and enhance overall scientific communication

Reviewer #2: None.

**Authors’ response:** Thank you for reviewing our manuscript. We appreciate the reviewer’s assessment and acknowledge that no additional comments or suggestions were provided.

Reviewer #3: There is a need for a few clarifications and corrections. I recommend minor revision to the manuscript before it is accepted for publication.

**Authors’ response:** Thank you very much for your constructive evaluation and recommendation for minor revision. We appreciate the reviewer’s careful assessment of our manuscript and have addressed all comments and suggestions by providing necessary clarifications, correcting identified issues, and improving the overall presentation of the manuscript. We believe that these revisions have strengthened the clarity, accuracy, and quality of the work.

**Summary and General Comments**

Use this section to provide overall comments, discuss strengths/weaknesses of the study, novelty, significance, general execution and scholarship. You may also include additional comments for the author, including concerns about dual publication, research ethics, or publication ethics. If requesting major revision, please articulate the new experiments that are needed.

Reviewer #1: The study entitled “Prevalence of Strongyloides stercoralis Infection among African Children: A Systematic Review and Meta-Analysis” has been submitted for peer review. It is a relevant, important, and necessary study, particularly with regard to infections caused by soil-transmitted helminths. However, some points need to be clarified.

**Authors’ response:** Thank you very much for your positive and constructive evaluation of our manuscript. We appreciate the reviewer’s recognition of the relevance, importance, and public health significance of assessing the prevalence of Strongyloides stercoralis infection among African children. We agree that several points required clarification, and we have carefully addressed all comments by revising the manuscript,

1. Why did the authors define a time period? The relevance of this information is not clear.

**Authors’ response:** Thank you for this important comment. We defined the search period to capture the most recent and relevant epidemiological evidence on Strongyloides stercoralis infection among African children while ensuring that the findings reflect current diagnostic approaches, surveillance practices, and public health contexts. We have clarified the rationale for the selected time period in the Materials and Methods section to improve transparency.

2. What is the relevance of including “analysis of blood samples”? Is this not a study focused solely on coproparasitological evaluation?

**Authors’ response:** Thank you for this important comment. We agree that the primary focus of this review was the coproparasitological evaluation of Strongyloides stercoralis infection among children. The mention of “analysis of blood samples” in the eligibility criteria was included because some studies may have used serological methods as part of Strongyloides diagnosis; however, this was not intended to imply that blood-based investigations were the main focus of the review

3. One of the objectives of the study is the analysis of parasitological methods, and this is precisely where an important weakness lies.

**Authors’ response: Th**ank you for this important comment. We agree that the assessment of diagnostic methods is a key objective of this review and that the original manuscript did not sufficiently emphasize the limitations associated with the available parasitological methods. We have revised the Results and Discussion sections to provide a more detailed evaluation of the diagnostic approaches used in the included studies, highlighting the differences in sensitivity among methods and the potential impact of relying predominantly on low-sensitivity techniques on the reported prevalence estimates. We have also clarified that variations in diagnostic methods represent an important source of heterogeneity and a limitation of the available evidence.

4. Why did the authors exclude the Kato-Katz method but not direct smear microscopy and formalin-ether concentration?

**Authors’ response:** Thank you for this important comment. The Kato-Katz method was excluded because it is primarily designed for the detection and quantification of intestinal schistosomes and soil-transmitted helminths with eggs that are readily detectable in stool, whereas Strongyloides stercoralis diagnosis depends on the detection of larvae, which are poorly detected by Kato-Katz due to the low and intermittent larval output in feces. Therefore, Kato-Katz has very limited sensitivity for diagnosing strongyloidiasis.

5. What is the relevance of the category “identification of studies via other methods” in Figure 1?

**Authors’ response:** Thank you for this comment. The “Identification of studies via other methods” pathway was included because the PRISMA 2020 flow diagram template provides this category to account for studies identified through sources other than electronic databases, such as citation searching or other supplementary methods. However, no studies were identified through these additional methods in our review. To avoid confusion, we have revised Figure 1 and removed this pathway, using the PRISMA 2020 flow diagram format appropriate for reviews based solely on database searches.

6. The discussion should be further developed, addressing clinical and policy implications. It is important to emphasize that the ages of the children vary, as do their lifestyles. The authors should reflect on and discuss this issue.

**Authors’ response:** Thank you for this important comment. We agree that the Discussion required further development regarding the clinical and policy implications of our findings. We have expanded the discussion to address how differences in children’s age groups, living conditions, environmental exposures, and lifestyles may influence the risk and distribution of S. stercoralis infection.

7. The study includes works that used different parasitological and molecular diagnostic techniques, which may introduce bias due to differences in diagnostic parameters among methods. Even though the authors acknowledge this in the discussion, it is important to critically reflect on the findings of this study. Does the reported prevalence truly reflect reality? Perhaps the authors should consider analyzing the techniques separately.

**Authors’ response:** Thank you for this important comment. We agree that differences in diagnostic techniques may introduce variability and potential bias in prevalence estimates because of differences in sensitivity and specificity among methods. However, the reported prevalence in this review reflects the epidemiological evidence available from the included studies and represents the burden of *S. stercoralis* infection detected using the diagnostic approaches applied in those settings. We acknowledge that conventional parasitological methods, such as direct smear microscopy and formalin-ether concentration, may underestimate the true prevalence due to their limited sensitivity. Nevertheless, these methods remain the most commonly used diagnostic approaches in many endemic settings and therefore provide important information on the currently documented burden of infection.

8. What is the interpretation of the fact that only eight African countries have studies reporting strongyloidiasis prevalence? Is there any geospatial relevance regarding the countries where these studies were conducted?

**Authors’ response:** Thank you for this important comment. We agree that the geographical distribution of available studies provides important context for interpreting the findings. The fact that prevalence data were available from only eight African countries highlights a substantial evidence gap and suggests that the reported burden may not fully represent the epidemiological situation across the continent. The absence of data from many African countries may reflect limited surveillance capacity, insufficient diagnostic resources, and under-recognition of strongyloidiasis rather than the absence of transmission.

Reviewer #2

Thank you for the opportunity to review manuscript PNTD-D-26-00795 by Hailu et al.
The manuscript authors present the findings from a systematic review and meta-analysis of S. stercoralis in African children. The results presented in the manuscript are relevant to inform programmatic interventions (preventive chemotherapy) to tackle STH and S. stercoralis in particular. Overall, the manuscript is well written; it requires extensive revisions to clarify a few aspects and improve its readability, but this does not in any way diminish the relevance of the findings presented.

**Authors’ response:** Thank you very much for your careful review and constructive feedback. We appreciate the reviewer’s recognition of the relevance of our systematic review and meta-analysis in informing programmatic interventions, including preventive chemotherapy strategies for *S. stercoralis* and soil-transmitted helminth control among children in Africa. We acknowledge the reviewer’s suggestions regarding the need for clarification and improvement of readability. We have carefully addressed all comments by revising the manuscript to improve the presentation of the methods,

1. Please review the text carefully to identify typos and rewrite sentences that don’t make sense due to grammatical mistakes throughout the document. Examples are:

a. Line 161: “Africa children …”, should be “African children …”

**Authors’ response:** Thank you very much for your careful review. Correction has been done.
b. Line 195: “For instance, the prevalence is relatively high in Ethiopia might be due to the application of a better sensitive diagnostic method …” should be “For instance, the relatively high prevalence in Ethiopia might be due to the application of more sensitive diagnostic methods”.

**Authors’ response:** Thank you very much for your careful review. Correction has been done (line 157)

c. Line 208: “The was also observed a variation in the study results …” should be “A variation in the study results was observed …”

**Authors’ response:** Thank you very much for your careful review. Correction has been done (line 170)

2. Authors should consider submitting the paper to English copy editing services to improve the readability of the document overall.

**Authors’ response:** Thank you very much for your careful review. English language correction has been done throughout the document.

3. The authors did not mention or comment anywhere in the manuscript that the World Health Organization has released the “WHO guideline on preventive chemotherapy for public health control of strongyloidiasis”. Authors should take this into consideration when making any recommendations on the next steps that should be taken by researchers or public health practitioners.

**Authors’ response:** Thank you very much for your careful review. Revision has been done on the recommendation (line 197-99)

Reviewer #3:

The manuscript fills an important public health gap, although there are some discrepancies in the interpretation and analysis of the results in the discussion, as well as some grammatical errors. I recommend minor revision.

**Authors’ response:** Thank you very much for your careful review and positive feedback. Revision has been done throughout the document.
