# Supplementary material for: Prevalence of Strongyloides stercoralis infection among African children: A systematic review and meta-analysis
Source: PLoS Negl Trop Dis. 2026 Oct 7;20(10):e0014759. doi: 10.1371/journal.pntd.0014759 (PMC13667923; doi:10.1371/journal.pntd.0014759)
Supplement: Peer review history

# Peer review: Decision Letter 0

**DOI:** 10.1371/journal.pntd.0014759.r001

**Authors:** Uriel Koziol, Eduardo José Lopes-Torres

7 Jul 2026

PNTD-D-26-00795

Prevalence of Strongyloides stercoralis Infection among African Children: A Systematic Review and Meta-Analysis

PLOS Neglected Tropical Diseases

Dear Dr. Hailu,

Thank you for submitting your manuscript to PLOS Neglected Tropical Diseases. After careful consideration, we feel that it has merit but does not fully meet PLOS Neglected Tropical Diseases's publication criteria as it currently stands. Therefore, we invite you to submit a revised version of the manuscript that addresses the points raised during the review process.

Please submit your revised manuscript within by Sep 05 2026 11:59PM. If you will need more time than this to complete your revisions, please reply to this message or contact the journal office at plosntds@plos.org. When you're ready to submit your revision, log on to https://www.editorialmanager.com/pntd/ and select the 'Submissions Needing Revision' folder to locate your manuscript file.

Please include the following items when submitting your revised manuscript:

* A letter that responds to each point raised by the editor and reviewer(s). You should upload this letter as a separate file labeled 'Response to Reviewers'. This file does not need to include responses to any formatting updates and technical items listed in the 'Journal Requirements' section below.

* A marked-up copy of your manuscript that highlights changes made to the original version. You should upload this as a separate file labeled 'Revised Manuscript with Track Changes'.

* An unmarked version of your revised paper without tracked changes. You should upload this as a separate file labeled 'Manuscript'.

If you would like to make changes to your financial disclosure, competing interests statement, or data availability statement, please make these updates within the submission form at the time of resubmission. Guidelines for resubmitting your figure files are available below the reviewer comments at the end of this letter.

As the corresponding author, your ORCID iD is verified in the submission system and will appear in the published article. PLOS supports the use of ORCID, and we encourage all coauthors to register for an ORCID iD and use it as well. Please encourage your coauthors to verify their ORCID iD within the submission system before final acceptance, as unverified ORCID iDs will not appear in the published article. Only the individual author can complete the verification step; PLOS staff cannot verify ORCID iDs on behalf of authors.

We look forward to receiving your revised manuscript.

Kind regards,

Eduardo José Lopes-Torres, Ph.D.

Academic Editor

PLOS Neglected Tropical Diseases

Uriel Koziol

Section Editor

PLOS Neglected Tropical Diseases

Shaden Kamhawi

co-Editor-in-Chief

PLOS Neglected Tropical Diseases

orcid.org/0000-0003-4304-636XX

Paul Brindley

co-Editor-in-Chief

PLOS Neglected Tropical Diseases

orcid.org/0000-0003-1765-0002

**Additional Editor Comments (if provided):**

**Journal Requirements:**

If the reviewer comments include a recommendation to cite specific previously published works, please review and evaluate these publications to determine whether they are relevant and should be cited. There is no requirement to cite these works unless the editor has indicated otherwise.

1) We noticed that you used the phrase 'unpublished data' in the manuscript. We do not allow these references, as the PLOS data access policy requires that all data be either published with the manuscript or made available in a publicly accessible database. Please amend the supplementary material to include the referenced data or remove the references.

2) Please upload all main figures as separate Figure files in .tif or .eps format. For more information about how to convert and format your figure files please see our guidelines:

https://journals.plos.org/plosntds/s/figures

3) Please upload a copy of Figure Fig 1-4 which you refer to in your text on pages 4, 7, 12, and 14. Or, if the figure is no longer to be included as part of the submission please remove all reference to it within the text.

4) We have noticed that you have uploaded Supporting Information files, but you have not included a list of legends. Please add a full list of legends for your Supporting Information files after the references list.

5) As required by our policy on Data Availability, please ensure your manuscript or supplementary information includes the following:

- A numbered table of all studies identified in the literature search, including those that were excluded from the analyses.

- For every excluded study, the table should list the reason(s) for exclusion.

- If any of the included studies are unpublished, include a link (URL) to the primary source or detailed information about how the content can be accessed.

- A table of all data extracted from the primary research sources for the systematic review and/or meta-analysis. The table must include the following information for each study:

- Name of data extractors and date of data extraction

- Confirmation that the study was eligible to be included in the review.

- All data extracted from each study for the reported systematic review and/or meta-analysis that would be needed to replicate your analyses.

- If data or supporting information were obtained from another source (e.g. correspondence with the author of the original research article), please provide the source of data and dates on which the data/information were obtained by your research group.

- If applicable for your analysis, a table showing the completed risk of bias and quality/certainty assessments for each study or outcome.  Please ensure this is provided for each domain or parameter assessed. For example, if you used the Cochrane risk-of-bias tool for randomized trials, provide answers to each of the signalling questions for each study. If you used GRADE to assess certainty of evidence, provide judgements about each of the quality of evidence factor. This should be provided for each outcome.

- An explanation of how missing data were handled.

This information can be included in the main text, supplementary information, or relevant data repository. Please note that providing these underlying data is a requirement for publication in this journal, and if these data are not provided your manuscript might be rejected.

6) We have amended your Competing Interest statement to comply with journal style. We kindly ask that you double check the statement and let us know if anything is incorrect.

**Reviewers' Comments:**

Reviewer's Responses to Questions

**Key Review Criteria Required for Acceptance?**

As you describe the new analyses required for acceptance, please consider the following:

**Methods**

-Are the objectives of the study clearly articulated with a clear testable hypothesis stated?

-Is the study design appropriate to address the stated objectives?

-Is the population clearly described and appropriate for the hypothesis being tested?

-Is the sample size sufficient to ensure adequate power to address the hypothesis being tested?

-Were correct statistical analysis used to support conclusions?

-Are there concerns about ethical or regulatory requirements being met?

Reviewer #1: -Are the objectives of the study clearly articulated with a clear testable hypothesis stated? No

-Is the study design appropriate to address the stated objectives? Yes

-Is the population clearly described and appropriate for the hypothesis being tested? No

-Is the sample size sufficient to ensure adequate power to address the hypothesis being tested? Yes

-Were correct statistical analysis used to support conclusions? Yes

-Are there concerns about ethical or regulatory requirements being met? No

Reviewer #2: The methods are described clearly in the M&M section, however, there are some issues. Here are some things to take into consideration:

1. On Figure 1 “Overview of search methods articles inclusion and exclusion criteria”. The right-hand side panel, where “Identification of studies via other methods is mentioned” does not add anything to the manuscript, especially since no studies were selected for assessment in this way. Please use the appropriate PRISMA Flow Diagram for this review.

2. Still on Figure 1, what was the total number of records (651?) that were retrieved from the 4 databases, from which 100 were duplicates, 39 records marked as ineligible by automation tools and the 3 removed for other reasons?

3. Please describe in detail within the Materials and Methods which automation tools were used before the initial screening of records. Questions arising from this include: Was this an artificial intelligence tool? What were the criteria/thresholds for these tools to decide which records to keep or not? And were the records excluded by these tools reviewed to ensure relevant studies were not mistakenly removed?

4. Still on Figure 1, there are some asterisk markings (“*”), which I presume would indicate the need for footnotes or something similar, which are not presented within the figure itself or on its caption.

5. Figure 1 in general, needs to be revised and improved very carefully. There are stages where it just states “Records excluded” without providing the reason, or even “Records screened (n=510)” without stating what was used to screen (e.g., title and abstract, etc.).

Reviewer #3: The study has a clear and appropriate study design. The methods used to conduct the systematic review and meta-analysis are appropriate and adequate.

**********

**Results**

-Does the analysis presented match the analysis plan?

-Are the results clearly and completely presented?

-Are the figures (Tables, Images) of sufficient quality for clarity?

Reviewer #1: - Does the analysis presented match the analysis plan? There are issues with conducting the analyses.

-Are the results clearly and completely presented? The results are well presented, but they have some problems.

-Are the figures (Tables, Images) of sufficient quality for clarity? Yes

Reviewer #2: 1. Line 144 and Table 1: please include the meaning of each and every abbreviation used in Table, since these are included only for DSM, FEC, BCT and APC, but are missing for qPCR, PCR, RT-PCR, STST. Also, please review the way the names of countries in the table are spelled to correct typos: “Co^te d’Ivory” should either be “Côte d’Ivoire” or “Ivory Coast”.

2. Please be careful and consistent with the abbreviation for real-time Polymerase Chain Reaction. In some instances “qPCR” is used, and in others, “RT-PCR”. However, the latter can be confused with reverse-transcriptase Polymerase chain reaction, which is a different method. Please verify this throughout the entire manuscript.

Reviewer #3: Most of the results are clearly presented. However, there is a discrepancy in some interpretations between the results and the discussion sections. The discrepancies are stated in the attached report. There are a few grammatical errors as well.

The authors did not acknowledge the limitations of their review, noting that the studies varied in their designs, geographic regions, and diagnostic methods.

**********

**Conclusions**

-Are the conclusions supported by the data presented?

-Are the limitations of analysis clearly described?

-Do the authors discuss how these data can be helpful to advance our understanding of the topic under study?

-Is public health relevance addressed?

Reviewer #1: -Are the conclusions supported by the data presented? Yes

-Are the limitations of analysis clearly described? No

-Do the authors discuss how these data can be helpful to advance our understanding of the topic under study? No

-Is public health relevance addressed? Yes!

Reviewer #2: Yes, the manuscript’s conclusions are mostly supported by the data presented, and limitations are also described, however, there are some issues in both the discussion and conclusions.

1. In the last paragraph of the Discussion (lines 215-217), the authors mention that “Additional limitations included restriction to English-language publications, the possibility of missing unpublished studies or grey literature, and the absence of automation tools or machine learning-assisted screening methods during the study selection process”. However, as mentioned in earlier comments, in Figure 1, authors have indicated that 38 records were excluded from the analysis as “Records marked as ineligible by automation tools (n = 38)”. Please clarify whether automated tools were used or not, and describe that appropriately in the Materials and Methods section of the manuscript.

2. The conclusion states (lines 224-226) that “Thus, we urge researchers to strive towards establishing standard procedures for diagnosing strongyloidiasis and write policy briefs that promote the inclusion of S. stercoralis in the soil-transmitted helminths control and prevention package.”. However, the “WHO guideline on preventive chemotherapy for public health control of strongyloidiasis” in July 2024; therefore, authors should take this into consideration when making such a statement.

Reviewer #3: The conclusion represents the findings in the manuscript. The authors provided an important policy recommendation of including S.stercoralis infection in the soil-transmitted helminths control and prevention package.

**********

**Editorial and Data Presentation Modifications?**

Use this section for editorial suggestions as well as relatively minor modifications of existing data that would enhance clarity. If the only modifications needed are minor and/or editorial, you may wish to recommend “Minor Revision” or “Accept”.

Reviewer #1: The authors should revise the English.

Reviewer #2: None.

Reviewer #3: There is a need for a few clarifications and corrections. I recommend minor revision to the manuscript before it is accepted for publication.

**********

**Summary and General Comments**

Use this section to provide overall comments, discuss strengths/weaknesses of the study, novelty, significance, general execution and scholarship. You may also include additional comments for the author, including concerns about dual publication, research ethics, or publication ethics. If requesting major revision, please articulate the new experiments that are needed.

Reviewer #1: The study entitled “Prevalence of Strongyloides stercoralis Infection among African Children: A Systematic Review and Meta-Analysis” has been submitted for peer review. It is a relevant, important, and necessary study, particularly with regard to infections caused by soil-transmitted helminths. However, some points need to be clarified.

1. Why did the authors define a time period? The relevance of this information is not clear.

2. What is the relevance of including “analysis of blood samples”? Is this not a study focused solely on coproparasitological evaluation?

3. One of the objectives of the study is the analysis of parasitological methods, and this is precisely where an important weakness lies.

4. Why did the authors exclude the Kato-Katz method but not direct smear microscopy and formalin-ether concentration?

5. What is the relevance of the category “identification of studies via other methods” in Figure 1?

6. The discussion should be further developed, addressing clinical and policy implications. It is important to emphasize that the ages of the children vary, as do their lifestyles. The authors should reflect on and discuss this issue.

7. The study includes works that used different parasitological and molecular diagnostic techniques, which may introduce bias due to differences in diagnostic parameters among methods. Even though the authors acknowledge this in the discussion, it is important to critically reflect on the findings of this study. Does the reported prevalence truly reflect reality? Perhaps the authors should consider analyzing the techniques separately.

8. What is the interpretation of the fact that only eight African countries have studies reporting strongyloidiasis prevalence? Is there any geospatial relevance regarding the countries where these studies were conducted?

Reviewer #2: Dear Editors,

Thank you for the opportunity to review manuscript PNTD-D-26-00795 by Hailu et al.

The manuscript authors present the findings from a systematic review and meta-analysis of Strongyloides stercoralis in African children. The results presented in the manuscript are relevant to inform programmatic interventions (preventive chemotherapy) to tackle STH and S. stercoralis in particular. Overall, the manuscript is well written; it requires extensive revisions to clarify a few aspects and improve its readability, but this does not in any way diminish the relevance of the findings presented.

1. Please review the text carefully to identify typos and rewrite sentences that don’t make sense due to grammatical mistakes throughout the document. Examples are:

a. Line 161: “Africa children …”, should be “African children …”

b. Line 195: “For instance, the prevalence is relatively high in Ethiopia might be due to the application of a better sensitive diagnostic method …” should be “For instance, the relatively high prevalence in Ethiopia might be due to the application of more sensitive diagnostic methods”.

c. Line 208: “The was also observed a variation in the study results …” should be “A variation in the study results was observed …”

2. Authors should consider submitting the paper to English copy editing services to improve the readability of the document overall.

3. The authors did not mention or comment anywhere in the manuscript that the World Health Organization has released the “WHO guideline on preventive chemotherapy for public health control of strongyloidiasis”. Authors should take this into consideration when making any recommendations on the next steps that should be taken by researchers or public health practitioners.

Reviewer #3: The manuscript fills an important public health gap, although there are some discrepancies in the interpretation and analysis of the results in the discussion, as well as some grammatical errors. I recommend minor revision.

**********

PLOS authors have the option to publish the peer review history of their article (what does this mean?). If published, this will include your full peer review and any attached files.

If you choose “no”, your identity will remain anonymous but your review may still be made public.

**Do you want your identity to be public for this peer review?**  For information about this choice, including consent withdrawal, please see our Privacy Policy.

Reviewer #1: No

Reviewer #2: No

Reviewer #3: **Yes:**  Salwa Shehu Dawaki

[NOTE: If reviewer comments were submitted as an attachment file, they will be attached to this email and accessible via the submission site. Please log into your account, locate the manuscript record, and check for the action link "View Attachments". If this link does not appear, there are no attachment files.]

**Figure resubmission:**

While revising your submission, we strongly recommend that you use PLOS’s NAAS tool (https://ngplosjournals.pagemajik.ai/artanalysis) to test your figure files. NAAS can convert your figure files to the TIFF file type and meet basic requirements (such as print size, resolution), or provide you with a report on issues that do not meet our requirements and that NAAS cannot fix.

After uploading your figures to PLOS’s NAAS tool - https://ngplosjournals.pagemajik.ai/artanalysis, NAAS will process the files provided and display the results in the "Uploaded Files" section of the page as the processing is complete. If the uploaded figures meet our requirements (or NAAS is able to fix the files to meet our requirements), the figure will be marked as "fixed" above. If NAAS is unable to fix the files, a red "failed" label will appear above. When NAAS has confirmed that the figure files meet our requirements, please download the file via the download option, and include these NAAS processed figure files when submitting your revised manuscript.

**Reproducibility:**

To enhance the reproducibility of your results, we recommend that authors of applicable studies deposit laboratory protocols in protocols.io, where a protocol can be assigned its own identifier (DOI) such that it can be cited independently in the future. Additionally, PLOS ONE offers an option to publish peer-reviewed clinical study protocols. Read more information on sharing protocols at https://plos.org/protocols?utm_medium=editorial-email&utm_source=authorletters&utm_campaign=protocols

Attachment Submitted filename: REVIEW_S.s_Africa_PNTD-D-26-00795.pdf

# Author comment: Author response to Decision Letter 1

**DOI:** 10.1371/journal.pntd.0014759.r002

22 Jul 2026

Attachment Submitted filename: Authors response to editor and reviewers comments.docx

# Peer review: Decision Letter 1

**DOI:** 10.1371/journal.pntd.0014759.r003

**Authors:** Uriel Koziol, Eduardo José Lopes-Torres

17 Sep 2026

Dear Dr Hailu,

We are pleased to inform you that your manuscript 'Prevalence of Strongyloides stercoralis Infection among African Children: A Systematic Review and Meta-Analysis' has been provisionally accepted for publication in PLOS Neglected Tropical Diseases.

Before your manuscript can be formally accepted you will need to complete some formatting changes, which you will receive in a follow up email. A member of our team will be in touch with a set of requests.

Please note that your manuscript will not be scheduled for publication until you have made the required changes, so a swift response is appreciated.

IMPORTANT: The editorial review process is now complete. PLOS will only permit corrections to spelling, formatting or significant scientific errors from this point onwards. Requests for major changes, or any which affect the scientific understanding of your work, will cause delays to the publication date of your manuscript.

Should you, your institution's press office or the journal office choose to press release your paper, you will automatically be opted out of early publication. We ask that you notify us now if you or your institution is planning to press release the article. All press must be co-ordinated with PLOS.

Thank you again for supporting Open Access publishing; we are looking forward to publishing your work in PLOS Neglected Tropical Diseases.

Best regards,

Eduardo José Lopes-Torres, Ph.D.

Academic Editor

PLOS Neglected Tropical Diseases

Uriel Koziol

Section Editor

PLOS Neglected Tropical Diseases

Shaden Kamhawi

co-Editor-in-Chief

PLOS Neglected Tropical Diseases

orcid.org/0000-0003-4304-636XX

Paul Brindley

co-Editor-in-Chief

PLOS Neglected Tropical Diseases

orcid.org/0000-0003-1765-0002

***********************************************************

Reviewer's Responses to Questions

**Key Review Criteria Required for Acceptance?**

As you describe the new analyses required for acceptance, please consider the following:

**Methods**

-Are the objectives of the study clearly articulated with a clear testable hypothesis stated?

-Is the study design appropriate to address the stated objectives?

-Is the population clearly described and appropriate for the hypothesis being tested?

-Is the sample size sufficient to ensure adequate power to address the hypothesis being tested?

-Were correct statistical analysis used to support conclusions?

-Are there concerns about ethical or regulatory requirements being met?

Reviewer #1: -Are the objectives of the study clearly articulated with a clear testable hypothesis stated? yes

-Is the study design appropriate to address the stated objectives? yes

-Is the population clearly described and appropriate for the hypothesis being tested? yes

-Is the sample size sufficient to ensure adequate power to address the hypothesis being tested? yes

-Were correct statistical analysis used to support conclusions? yes

-Are there concerns about ethical or regulatory requirements being met? yes

Reviewer #2: Despite the improvements and authors’ responses, issues with Figure 1 persist. Namely:

1. Authors should use the “PRISMA 2020 flow diagram for new systematic reviews which included searches of databases and registers only”, instead of the “PRISMA 2020 flow diagram for new systematic reviews which included searches of databases, registers and other sources”. This will solve some of the issues raised during the first round of review.

2. Polish the information inside each box, and remove unnecessary text. For example, in the top left box about identification of studies, text reads as “Records identified from: Databases (n=4), Registers (n=0), (n=651)”. This raises the question of whether there was a third source or not; do the 651 records correspond to the studies retrieved from the “Registers”? Please clarify this.

3. Authors’ response states that “Thank you for this important comment. We apologize for the confusion caused by the incorrect information in the PRISMA flow diagram. No automation tools or artificial intelligence-assisted screening methods were used during the study selection process. The statement "Records marked as ineligible by automation tools (n = 38)" was included in error and has been corrected in the revised Figure 1. The 38 records were manually screened based on their titles and abstracts according to the predefined eligibility criteria and were excluded because they did not meet the inclusion criteria. We have revised the Materials and Methods section accordingly to accurately describe the manual screening process and ensure consistency throughout the manuscript.”, yet Figure 1 still states “Records marked as ineligible by automation tools (n=38)”.

Reviewer #3: The manuscript has been submitted for review, and the authors have revised it to address all reviewer recommendations. It is clearer and more clearly articulated.

**********

**Results**

-Does the analysis presented match the analysis plan?

-Are the results clearly and completely presented?

-Are the figures (Tables, Images) of sufficient quality for clarity?

Reviewer #1: -Does the analysis presented match the analysis plan? yes

-Are the results clearly and completely presented? yes

-Are the figures (Tables, Images) of sufficient quality for clarity? yes

Reviewer #2: Comments raised in the first round of revision have been addressed appropriately.

Reviewer #3: Yes, it is well presented.

**********

**Conclusions**

-Are the conclusions supported by the data presented?

-Are the limitations of analysis clearly described?

-Do the authors discuss how these data can be helpful to advance our understanding of the topic under study?

-Is public health relevance addressed?

Reviewer #1: -Are the conclusions supported by the data presented? yes

-Are the limitations of analysis clearly described? reasonably

-Do the authors discuss how these data can be helpful to advance our understanding of the topic under study? yes

-Is public health relevance addressed? YES!

Reviewer #2: Comments raised in the first round of revision have been addressed appropriately.

Reviewer #3: The conclusions support the data presented, and the limitations clearly stated

**********

**Editorial and Data Presentation Modifications?**

Use this section for editorial suggestions as well as relatively minor modifications of existing data that would enhance clarity. If the only modifications needed are minor and/or editorial, you may wish to recommend “Minor Revision” or “Accept”.

Reviewer #1: no

Reviewer #2: Figure 1 needs to be modified according to comments included in other parts of this revision to make it clear and the study reproducible.

Reviewer #3: Accept

**********

**Summary and General Comments**

Use this section to provide overall comments, discuss strengths/weaknesses of the study, novelty, significance, general execution and scholarship. You may also include additional comments for the author, including concerns about dual publication, research ethics, or publication ethics. If requesting major revision, please articulate the new experiments that are needed.

Reviewer #1: I appreciate the opportunity to review the revised version of the manuscript entitled “Prevalence of Strongyloides stercoralis Infection among African Children: A Systematic Review and Meta-Analysis.” The topic is relevant and aligns well with the scope of the journal, particularly given the ongoing challenges in estimating the true burden of strongyloidiasis in endemic regions.

The authors have addressed several of the reviewers’ and editor’s comments. Overall, the points previously raised were considered, although some were addressed in a somewhat superficial manner; nevertheless, the responses remain acceptable.

I consider that the manuscript is suitable for publication.

Finally, I would like to highlight that the presentation of information regarding parasitological techniques is adequate. Although the discussion on this aspect is not extensively developed, it is still presented in an acceptable manner within the scope of the manuscript.

Reviewer #2: None.

Reviewer #3: The authors have revised the manuscript very well.

**********

PLOS authors have the option to publish the peer review history of their article (what does this mean?). If published, this will include your full peer review and any attached files.

If you choose “no”, your identity will remain anonymous but your review may still be made public.

**Do you want your identity to be public for this peer review?**  For information about this choice, including consent withdrawal, please see our Privacy Policy.

Reviewer #1: No

Reviewer #2: No

Reviewer #3: **Yes:**  Salwa Shehu Dawaki

# Editor report: Acceptance letter

**DOI:** 10.1371/journal.pntd.0014759.r004

**Authors:** Uriel Koziol, Eduardo José Lopes-Torres

Dear Dr Hailu,

We are delighted to inform you that your manuscript, "Prevalence of Strongyloides stercoralis Infection among African Children: A Systematic Review and Meta-Analysis," has been formally accepted for publication in PLOS Neglected Tropical Diseases.

We have now passed your article onto the PLOS Production Department who will complete the rest of the publication process. All authors will receive a confirmation email upon publication.

The corresponding author will soon be receiving a typeset proof for review, to ensure errors have not been introduced during production. Please review the PDF proof of your manuscript carefully, as this is the last chance to correct any scientific or type-setting errors. Please note that major changes, or those which affect the scientific understanding of the work, will likely cause delays to the publication date of your manuscript. Note: Proofs for Front Matter articles (Editorial, Viewpoint, Symposium, Review, etc...) are generated on a different schedule and may not be made available as quickly.

Soon after your final files are uploaded, the early version of your manuscript will be published online unless you opted out of this process. The date of the early version will be your article's publication date. The final article will be published to the same URL, and all versions of the paper will be accessible to readers.

For Research Articles, you will receive an invoice from PLOS for your publication fee after your manuscript has reached the completed accept phase. If you receive an email requesting payment before acceptance or for any other service, this may be a phishing scheme. Learn how to identify phishing emails and protect your accounts at https://explore.plos.org/phishing.

Thank you again for supporting open-access publishing; we are looking forward to publishing your work in PLOS Neglected Tropical Diseases.

Best regards,

Shaden Kamhawi

co-Editor-in-Chief

PLOS Neglected Tropical Diseases

Paul Brindley

co-Editor-in-Chief

PLOS Neglected Tropical Diseases
